# Data envelopment analysis for scale elasticity measurement in the stochastic case: with an application to Indian banking

**DOI:** 10.1186/s40854-022-00447-1

**Published:** 2023-01-16

**Authors:** Alireza Amirteimoori, Biresh K. Sahoo, Saber Mehdizadeh

**Affiliations:** 1 Faculty of Engineering & Natural Sciences, Istinye University, Istanbul, Turkey; 2 Xavier Institute of Management, XIM University, Bhubaneswar, 7571013 India

**Keywords:** Data envelopment analysis, Stochastic data envelopment analysis, Technical efficiency, Returns to scale, Economies of scale, Scale elasticity, Indian banking, Econometrics, Economics

## Abstract

In the nonparametric data envelopment analysis literature, scale elasticity is evaluated in two alternative ways: using either the technical efficiency model or the cost efficiency model. This evaluation becomes problematic in several situations, for example (a) when input proportions change in the long run, (b) when inputs are heterogeneous, and (c) when firms face *ex-ante* price uncertainty in making their production decisions. To address these situations, a scale elasticity evaluation was performed using a value-based cost efficiency model. However, this alternative value-based scale elasticity evaluation is sensitive to the uncertainty and variability underlying input and output data. Therefore, in this study, we introduce a stochastic cost-efficiency model based on chance-constrained programming to develop a value-based measure of the scale elasticity of firms facing data uncertainty. An illustrative empirical application to the Indian banking industry comprising 71 banks for eight years (1998–2005) was made to compare inferences about their efficiency and scale properties. The key findings are as follows: First, both the deterministic model and our proposed stochastic model yield distinctly different results concerning the efficiency and scale elasticity scores at various tolerance levels of chance constraints. However, both models yield the same results at a tolerance level of 0.5, implying that the deterministic model is a special case of the stochastic model in that it reveals the same efficiency and returns to scale characterizations of banks. Second, the stochastic model generates higher efficiency scores for inefficient banks than its deterministic counterpart. Third, public banks exhibit higher efficiency than private and foreign banks. Finally, public and old private banks mostly exhibit either decreasing or constant returns to scale, whereas foreign and new private banks experience either increasing or decreasing returns to scale. Although the application of our proposed stochastic model is illustrative, it can be potentially applied to all firms in the information and distribution-intensive industry with high fixed costs, which have ample potential for reaping scale and scope benefits.

## Introduction

Performance analysis of multi-product firms has been conducted using several economic concepts, such as economies of scale (returns to scale), economies of scope, and marginal rates of technical substitutions. However, this study concentrates on the determination and measurement of returns to scale, as it has important implications for policy design and industry regulation. Scale elasticity is a quantitative measure of the returns-to-scale characterization of a firm^1^ operating on the production frontier, and is used to determine improvement or deterioration in productivity by resizing their scales of operation.

In the literature, there are two analytical approaches to the empirical estimation of scale elasticity: the neoclassical and axiomatic approaches (Färe et al. 1988). While the former is usually estimated using parametric techniques such as stochastic frontier analysis (SFA), the latter is estimated nonparametric via data envelopment analysis (DEA). However, DEA^2^ has distinct advantages over stochastic frontier estimation (Sahoo and Tone 2022). First, DEA avoids the choice of specific functional forms and the choice of the stochastic structure, which the stochastic frontier approach suffers from due to which it can confound the effects of misspecification of functional form with scale economies (Fusco et al. 2018; Sahoo and Acharya 2010; Sahoo et al. 2017; Sahoo and Gstach 2011). Second, contrary to the general belief, DEA is a full-fledged statistical methodology based on the characterization of firm efficiency as a stochastic variable. DEA estimators have desirable properties and provide a basis for constructing a wide range of formal statistical tests (Banker 1993; Banker and Natarajan 2004; Banker et al. 2022). Third, DEA reflects individual firm efficiency or inefficiency, which is particularly convenient for managerial decision making.

In the DEA literature, both qualitative and quantitative approaches are used to evaluate the returns-to-scale characterization of firms. While the former deals with the identification of the types of returns to scale (i.e., increasing, decreasing, or constant), the latter deals with the computation of scale elasticity (Førsund 1996; Sahoo et al. 1999, 2012; Tone and Sahoo 2003, 2004; Podinovski et al. 2009; Sahoo and Sengupta 2014; Sahoo and Tone 2015). Most studies that apply bespoke computational methods to evaluate scale economies are confined to specific DEA technologies under the constant returns to scale (CRS) specification of Charnes et al. (1978) and the variable returns to scale (VRS) specification of Banker et al. (1984). However, there is a large class of polyhedral technologies that include CRS and VRS technologies with production trade-offs and weight restrictions (Tone 2001; Atici et al. 2015; Podinovski 2004a; 2007, 2015, 2016), and weak and managerially disposable technologies (Kuosmanen 2005; Kuosmanen and Podinovski. 2009), hybrid returns to scale (Podinovski 2004b), technology with multiple component processes (Cook and Zhu 2011; Cherchye et al. 2013, 2015, 2016), and network technologies (Sahoo et al. 2014a, b), etc. See Podinovski et al. (2016) for a discussion of scale elasticity evaluation using a more general methodology.

Scale elasticity evaluation was also performed using the cost efficiency model of Färe et al. (1985) (Sueyoshi 1997; Tone and Sahoo 2005, 2006). The use of this model requires that input prices are exogenously given and measured with full certainty. However, in real-life situations, input prices are not exogenous but vary according to the actions of firms (Sahoo and Tone 2013). In addition, firms often face *ex-ante* price uncertainty^3^ when making production decisions (Camanho and Dyson 2005). Furthermore, input prices are synthetically constructed and hence represent average prices. Since decisions are made at the margin, the measure of allocative efficiency based on average prices can be distorted (Fukuyama and Weber 2008). Finally, the cost efficiency measure of Färe et al. (1985) reflects only input inefficiencies, but not market (price) inefficiencies (Camanho and Dyson 2008).^4^ Therefore, to account for both input and market inefficiencies, Tone’s (2002) alternative cost-efficiency model can be used. Furthermore, the use of this value-based technology is more appropriate for computing the scale elasticity scores of real-life firms in several situations, for example (a) when input proportions change in the long run, (b) when inputs are heterogeneous, and (c) as the cost can be more easily related to economies of scale and size-specific costs due to indivisibility.

Scale elasticity evaluation in DEA has thus far been made using only deterministic technologies. These technologies were developed based on the premise that the inputs and outputs are precisely measured. However, in real-life situations, general production processes are often stochastic,^5^ and the concepts of efficiency and scale elasticity are inextricably related to how firms deal with uncertainty underlying stochastic inputs and outputs. Therefore, when there are variations in inputs and outputs owing to uncertainty, the evaluation of efficiency and scale elasticity in a deterministic DEA setting becomes sensitive to such variations. Consequently, one expects to observe whether the efficiency and returns-to-scale characteristics of firms are subject to change in this stochastic environment.

To account for random variations in inputs and outputs underlying any production process, several authors (Banker and Morey 1986; Cooper et al. 2004, 2011, 1996, 1998; Jess et al. 2001; Lamb and Tee 2012; Olesen 2006; Sengupta 1990) have considered various approaches in DEA setting to compute efficiency. Sengupta (1982) and Cooper et al. (1996) were the first to introduce the theory of chance constraints in DEA to formulate an efficiency evaluation for firms. This formulation was later extended by several scholars (Sengupta and Sfeir 1988; Land et al. 1993, 1994; Li 1995a, b, 1998; Olesen and Petersen 1995, 2000; Grosskopf 1996; Morita and Seiford 1999; Huang and Li 2001; Kao and Liu 2014; Wei et al. 2014). Cooper et al. (2011), Simar and Wilson (2015), and Olesen and Petersen (2016) provided extensive reviews of the development of various stochastic DEA models.

To the best of our knowledge, no studies in the DEA literature has dealt with the estimation of scale elasticity using a technology setup that allows such stochastic variations in the data. Therefore, in this study, we focus on the estimation of scale elasticity when inputs and outputs are uncertain in stochastic form. The existence of random changes in the data permits the prediction of changes in the inputs and outputs. Because deterministic programs are sensitive to such variations, we need the technique of chance-constrained (CC) programming^6^ (see Charnes and Cooper (1959, 1962, 1963) for its early developments) because this method can potentially deal with the cases in which the constraints–the minimal (maximal) inputs (outputs)–are required to be no more (less) than the actual inputs (outputs) may be violated, but not too frequently. Although stochastic variation around the production function is allowed, most observations must fall below it.

To set up value-based measures of efficiency and scale elasticity, first, we formulate, using the CC-programming method, the stochastic version of the alternative cost efficiency model of Tone (2002), which is essentially a quadratic model. Second, we set up a deterministic equivalent of the stochastic quadratic model. Third, with the help of a one-factor assumption, we converted this deterministic quadratic model into a linear model using the goal programming theory of Charnes and Cooper (1977). Fourth, we solved this linear model to compute the value-based efficiency scores of firms at pre-determined tolerance levels of chance constraints. Finally, we set up the dual of this linear model to compute the value-based scale elasticity scores of firms at these tolerance levels.

The method employed to convert the quadratic model into a linear model is based on a simplified single-factor assumption, which has long been used in economics. This single-factor assumption is extremely convenient for finding solutions but requires some additional assumptions that the correlation coefficient between any two stochastic inputs/outputs and between input and output for any firm is always 1 (Kao and Liu 2019). Thus, there is a tradeoff between a quadratic (nonlinear) model, which is inconvenient to solve, and a linear model, which is easy to solve but requires some additional assumptions. However, we believe that this perfect correlation assumption between inputs/outputs is certainly important as it helps convert a quadratic model into a linear one, which not only removes the inconveniences associated with solving a quadratic model, but also helps compute the scale elasticity scores of firms using its dual.

To demonstrate the ready applicability of our proposed model, we exhibited an illustrative empirical application wherein we employ Indian banking data, which was used earlier by Sahoo and Tone (2009a, b). It is important to investigate the extent of economies of scale in the banking industry in general, and the banking industry of a fast-growing economy like India, in particular. The banking industry is technology-driven, and technical progress is scale augmenting (Berger 2003). The evaluation of scale economies in India’s banking industry during the post-reform period from 1998 to 2005 is considered important because this exercise has important implications for policy design and regulation due to changing regulatory environments and different ownership types (public, private, and foreign). The Indian financial sector had initially been operating in a closed and regulated environment until 1992 and then underwent a radical change during the nineties. To promote the efficiency and profitability of banks, in 1991–92 the Reserve Bank of India (RBI) initiated a process of liberalization through various reforms such as entry deregulation, branch de-licensing, deregulation of interest rates, allowing public banks to raise their equity in the capital markets, gradual reduction in cash reserve ratio, statutory liquidity ratio, and relaxation of several restrictions on the composition of their portfolios. All of these have given rise to heightened competitive pressure in the banking industry. In this scenario, we believe that banks were in the pursuit of enlarging their size using available scale economies to enhance their asset base and profit to create systemic financial efficiency and shareholder value. Furthermore, the introduction of several reform measures and technological advances has put the banking industry in a more challenging and volatile position, making the underlying bank production processes stochastic. In this environment, following Shiraz et al. (2018), stochasticity in the inputs and outputs is first introduced, and our proposed stochastic efficiency model is then applied to these stochastic input–output data to produce valid efficiency and scale elasticity estimates.

In our empirical illustration, we examine the efficiency and returns-to-scale characteristics of banking in India across three ownership types: public, private, and foreign. This enables us to investigate the economic linkage between ownership and efficiency performance by considering the property rights hypothesis (De Alessi 1980) and public choice theory (Levy 1987; Niskanen 1975). According to the property rights hypothesis, private enterprises should perform more efficiently than public enterprises because of the strong link between the markets for corporate control and the efficiency of private enterprises. While this argument may apply more to developed countries, testing for efficiency differentials across ownership types in the banking industry of a developing country such as India can yield insights into the success of the reform process.

Although our current empirical application to Indian banking is illustrative, our proposed chance-constraint efficiency model can potentially be applied to analyze the efficiency and scale properties of many real-life firms whose underlying production processes are stochastic. Examples of these firms can be found in many industries, for example agriculture, where unpredictability in weather makes the input–output relationship stochastic; manufacturing industries, where firms face considerable variation in the quality of their inputs and outputs produced; product development industries, where firms face uncertainty regarding their new designs; and high-technology industries, where firms face hyper (dynamic) completion in the new (Internet) economy.

**Fig. 1:**
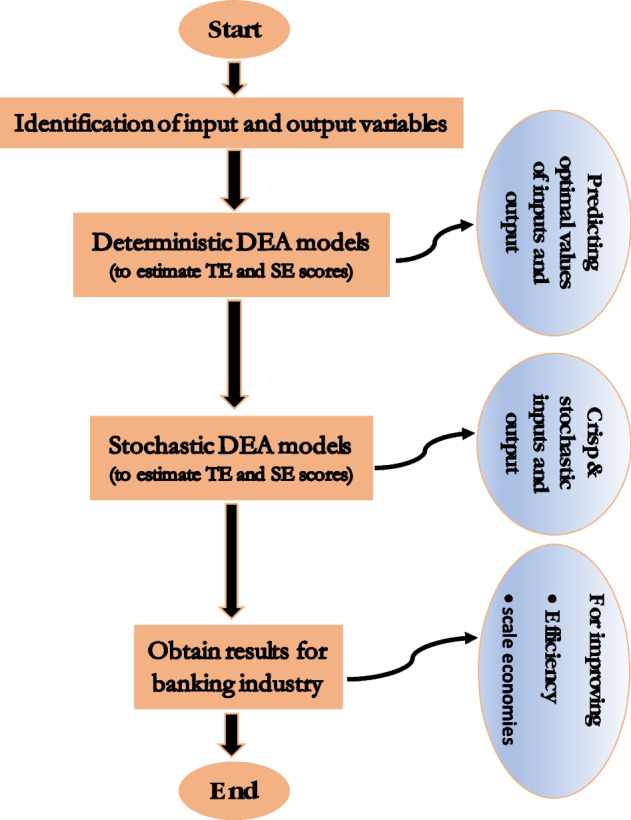
Conceptual framework

Finally, Fig. 1 depicts the conceptual framework of our investigation, highlighting both the methodological and applied approaches.

The remainder of the paper is organized as follows: “A value-based measure of efficiency and scale elasticity in the deterministic case” section discusses the evaluation of value-based scale elasticity measures in a deterministic DEA technology defined in the cost-output space. In “A value-based measure of TE and SE in the stochastic case” section, we first set up the stochastic version of Tone’s (2002) value-based cost efficiency model and then propose its transformation in a deterministic setting for the computations of value-based measures of efficiency and scale elasticity scores. “The illustrative empirical application” section demonstrates an illustrative empirical application of our proposed stochastic efficiency model to data on 71 banks in India. “Discussions” section presents a discussion of the results. Finally, we conclude with remarks in “Concluding remarks” section, followed by the limitations of our study and future recommendations.

## A value-based measure of efficiency and scale elasticity in the deterministic case

We suppose there are $n$ firms to be evaluated and each firm uses $m$ inputs to produce $s$ outputs. Let ${x}_{j} = {\left({x}_{1j} , . . . , {x}_{mj} \right)}^{T}\in {\mathbb{R}}_{\ge 0}^{m}$ and ${y}_{j}={\left({y}_{1j}, . . . , {y}_{sj} \right)}^{T}\in {\mathbb{R}}_{\ge 0}^{s}$ be the input and output vectors of firm$j$, respectively, ${w}_{j}=\left({w}_{1j}, . . . , {w}_{mj}\right)\in {\mathbb{R}}_{\ge 0}^{m}$ be its unit price vector, and $J$ be the index set of all firms, that is, $J = \{1, . . . , n\}$. We set up a value-based technology ${(T}^{V})$ set as

1 $${T}^{V}=\left\{{\left(c,y\right)}^{T} | \sum_{j=1}^{n}{\lambda }_{j}{c}_{j}\le c, \sum_{j=1}^{n}{\lambda }_{j}{y}_{rj}\ge {y}_{r} (\forall r), \sum_{j=1}^{n}{\lambda }_{j}=1, \lambda \ge 0\right\},$$

where ${c}_{j}=\sum_{i=1}^{m}{w}_{ij}{x}_{ij}.$ The ${\lambda =(\lambda }_{1}, {\lambda }_{2}, \dots , {\lambda }_{n})$ is an intensity vector of dimension* n*.

To produce the output vector $\beta {y}_{o}$ by firm $o$, its value-based measure of technical efficiency $(TE)$, $\overline{\alpha }\left(\beta \right)$ is defined as

2 $$\overline{\alpha }\left(\beta \right)=\mathrm{min} \left\{\alpha \right|\left(\alpha {c}_{o}, \beta {y}_{o}\right)\in {T}^{V}\}.$$

$\beta$ is a user-defined value that reflects the proportional output change, and $\overline{\alpha }(\beta )$ is defined for all $\beta \in [0,\widehat{\beta }]$, where $\widehat{\beta }$ is the largest proportion of the output vector ${y}_{o}$ found in units of technology ${T}^{V}$.

Based on the definition in (2), $\overline{\alpha }\left(\beta \right)$ can be determined using the following linear program (LP):

3 $$\overline{\alpha }\left(\beta \right)=\mathrm{min}\alpha$$

$$s.t.$$ $$\sum_{j=1}^{n}{\lambda }_{j}{c}_{j}\le \alpha {c}_{o},$$ $$\sum_{j=1}^{n}{\lambda }_{j}{y}_{rj}\ge \beta {y}_{ro} \left(\forall r\right),$$ $$\sum_{j=1}^{n}{\lambda }_{j}=1,$$ $${\lambda }_{j}\ge 0 \left(\forall j\right),\mathrm{ and }\alpha :\mathrm{free}.$$

Program (3) was used to identify explicit real peer units for each unit under evaluation. As previously stated, ${\lambda =(\lambda }_{1}, {\lambda }_{2}, \dots , {\lambda }_{n})$ is an intensity vector in which each ${\lambda }_{j}$ shows the effect of ${DMU}_{j}$ on constructing the peer unit for ${DMU}_{o}$ under evaluation.

The dual formulation of the program (3) can be set up as

4 $$\overline{\alpha }\left(\beta \right)=\mathrm{max} \beta \sum_{r=1}^{s}{u}_{r}{y}_{ro}+{\omega }_{0}$$

$$s. t.$$ $$\sum_{r=1}^{s}{u}_{r} {y}_{rj}-v{c}_{j}+{\omega }_{0}\le 0,$$ $$v{c}_{o}=1,$$ $$v,u\ge 0.$$

Based on program (4) for firm $o$, we derive its transformation function as

5 $$\psi \left(\overline{\alpha }(\beta ){c}_{o}, \beta {y}_{o}\right)=\beta \sum_{r=1}^{s}{u}_{r} {y}_{ro}-v\left(\overline{\alpha }\left(\beta \right){c}_{o}\right)+{\omega }_{0}=0,$$

We assume that the transformation function (5) is differentiable. Differentiating (5) with respect to $\beta$ yields.

$$\frac{\partial \psi \left(\overline{\alpha }(\beta ){c}_{o}, \beta {y}_{o}\right)}{\partial \beta }=\sum_{r=1}^{s}\frac{\partial \psi \left(\overline{\alpha }(\beta ){c}_{o}, \beta {y}_{o}\right)}{\partial \left(\beta {y}_{ro}\right)}{y}_{ro}+\frac{\partial \psi \left(\overline{\alpha }(\beta ){c}_{o}, \beta {y}_{o}\right)}{\partial \left(\overline{\alpha }(\beta ){c}_{o}\right)}{c}_{o}\frac{\partial \alpha }{\partial \beta }=0,$$

$$\Rightarrow \frac{\partial \overline{\alpha }(\beta )}{\partial \beta }=-\frac{\sum_{r=1}^{s}\frac{\partial \psi \left(\overline{\alpha }(\beta ){c}_{o}, \beta {y}_{o}\right)}{\partial \left(\beta {y}_{ro}\right)}{y}_{ro}}{\frac{\partial \psi \left(\overline{\alpha }(\beta ){c}_{o}, \beta {y}_{o}\right)}{\partial \left(\overline{\alpha }(\beta ){c}_{o}\right)}{c}_{o}}=-\frac{\sum_{r=1}^{s}{u}_{r}{y}_{ro}}{-v{c}_{o}}=\frac{\frac{\overline{\alpha }\left(\beta \right)-{\omega }_{o}}{\beta }}{v{c}_{o}}=\frac{\frac{\overline{\alpha }\left(\beta \right)-{\omega }_{o}}{\beta }}{1}=\frac{\overline{\alpha }\left(\beta \right)-{\omega }_{o}}{\beta }$$

In the spirit of Panzar and Willig (1977), Baumol et al. (1982), and Banker et al. (2004), we define the value-based scale elasticity $(SE)$ measure of firm $o$, $\varepsilon \left(\overline{\alpha }(\beta ){c}_{o}, \beta {y}_{o}\right)$ as the ratio of its marginal utilization of input $\frac{\partial \overline{\alpha }(\beta )}{\partial \beta }$ to its average utilization of input $\frac{\overline{\alpha }(\beta )}{\beta }$. In other words,

6 $$\varepsilon \left(\overline{\alpha }(\beta ){c}_{o}, \beta {y}_{o}\right)=\frac{\partial \overline{\alpha }(\beta )}{\partial \beta }\bullet \frac{\beta }{\overline{\alpha }\left(\beta \right)}=\frac{\overline{\alpha }\left(\beta \right)-{\omega }_{o}}{\beta }\bullet \frac{\beta }{\overline{\alpha }\left(\beta \right)}=1-\frac{{\omega }_{o}}{\overline{\alpha }\left(\beta \right)}$$

If the firm $o$ is technically efficient, we have $\beta =\overline{\alpha }\left(\beta \right)=1$ and $\varepsilon \left({c}_{o}, {y}_{o}\right)=1-{\omega }_{o}$.

In the immediately following section, we extend these results to the stochastic case.

## A value-based measure of ${\varvec{T}}{\varvec{E}}$ and ${\varvec{S}}{\varvec{E}}$ in the stochastic case

### Stochastic value-based ${\varvec{T}}{\varvec{E}}$ measure

Let ${\widetilde{x}}_{j}={\left({\widetilde{x}}_{1j}, {\widetilde{x}}_{2j},\dots ,{\widetilde{x}}_{Mj}\right)}^{T}\in {\mathbb{R}}_{\ge 0}^{m}$ and ${\widetilde{y}}_{j}={\left({\widetilde{y}}_{1j}, {\widetilde{y}}_{2j},\dots ,{\widetilde{y}}_{Sj}\right)}^{T}\in {\mathbb{R}}_{\ge 0}^{s}$ be the random input and output vectors of firm $j$, respectively. Furthermore, let $\left({\widetilde{x}}_{j}\right)=$ ${x}_{j}$, $E\left({\widetilde{y}}_{j}\right)={y}_{j}$, and all random inputs and outputs are jointly and normally distributed. The structure of our stochastic value-based technology set ${(T}^{V(S)})$ in the CC programming setup is related to the technology set employed in the BCC program. ${T}^{V(S)}$ is the union of confidence regions ${D}_{j}\left({1-\gamma }_{j}\right)$ with a probability level $(1-{\gamma }_{j})$ $(j=1,\dots ,n)$. In other words, ${T}^{V(S)}$ is the union of the input and output values that are probably observed for ${DMU}_{j} (j=1,\dots ,n)$ with probability ${1-\gamma }_{j}$. Using the axioms of free disposability, unbounded rays, convexity, minimal extrapolation, and inclusion of observations within confidence regions, ${T}^{V(S)}$ can be set up as follows:

7 $$\begin{aligned} T^{V\left( S \right)} = & \left\{ {\left( {c,y^{T} } \right)^{T} \in {\mathbb{R}}_{ + }^{1 + S} | \exists \left( {\hat{x}_{j}^{T} , \hat{y}_{j}^{T} } \right)^{T} \in D_{j} \left( {1 - \gamma_{j} } \right) {\text{and }} \lambda_{j} \ge 0, j \in \left\{ {1, \ldots ,n} \right\} {\text{such that}}} \right. \\ & \left. { \mathop \sum \limits_{j = 1}^{n} \lambda_{j} \hat{c}_{j} \le c\, {\text{and}}\; \mathop \sum \limits_{j = 1}^{n} \lambda_{j} \hat{y}_{rj} \ge y_{r} \left( {\forall r} \right), \mathop \sum \limits_{j = 1}^{n} \lambda_{j} = 1} \right\} \\ \end{aligned}$$

where ${\widehat{c}}_{j}=\sum_{i=1}^{m}{w}_{ij}{\widehat{x}}_{ij}$ and ${D}_{j}\left({1-\gamma }_{j}\right)$ is defined as follows:

8 $${D}_{j}\left({1-\gamma }_{j}\right)=\left\{{\left({\widehat{x}}^{T},{\widehat{y}}^{T}\right)}^{T}\in {\mathbb{R}}_{+}^{M+S}| \left[{\left(\widehat{x}-{x}_{j}\right)}^{T}, {\left(\widehat{y}-{y}_{j}\right)}^{T}\right]{\Lambda }_{\mathrm{j}}^{-1} {\left[{\left(\widehat{x}-{x}_{j}\right)}^{T}, {\left(\widehat{y}-{y}_{j}\right)}^{T}\right]}^{T}\le {c}_{j}^{2}\right\},$$

where ${\Lambda }_{j}^{-1}$ represents the inverse of the variance–covariance matrix of $({\widetilde{x}}_{j}, {\widetilde{y}}_{j})$. Moreover, ${c}_{j}$ is determined by ${\mathbb{P}}\left({\chi }_{M+S}^{2}\le {c}_{j}^{2}\right)=1-{\gamma }_{j}$, and ${\chi }_{M+S}^{2}$ is the Chi-square random variable with $M+S$ degrees of freedom.

Inspired by the deterministic value-based $TE$ measure $\overline{\alpha }\left(\beta \right)$ in program (3), we define its stochastic counterpart as the greatest possible radial contraction of ${\widetilde{c}}_{o}$ possible in ${T}^{V(S)}$ to produce $\beta {\widetilde{y}}_{o}$. Mathematically, the stochastic value-based $TE$ measure of firm $o$ can be computed by solving Eq. (9), where $\beta$ is a predefined parameter.

9 $$\overline{\alpha }\left(\beta \right)=\mathrm{min}\alpha$$

9.1 $$s.t.\;{\mathbb{P}}\left( {\mathop \sum \limits_{j = 1}^{n} \lambda_{j} \tilde{c}_{j} \le \alpha \tilde{c}_{o} } \right) \ge 1 - \gamma ,$$

9.2 $${\mathbb{P}}\left(\sum_{j=1}^{n}{\lambda }_{j}{\widetilde{y}}_{rj}\ge \beta {\widetilde{y}}_{ro}\right)\ge 1-\gamma (\forall r),$$

9.3 $$\sum_{j=1}^{n}{\lambda }_{j}=1,$$

$${\lambda }_{j}\ge 0 \left(\forall j\right),$$ and $$\alpha :\mathrm{free}.$$

In program (9), we used the “E-model” form of the marginal chance-constrained DEA program, which was introduced by Cooper et al. (2002) with the help of the BCC model of Banker et al. (1984). Therefore, we used two separate probability constraints: one for the input cost and the other for the set of outputs.

The objective of program (9) is to measure the stochastic value-based TE of any firm $o$ $(o=\mathrm{1,2},\dots ,n)$ because its input and outputs are all assumed to be stochastic. This is achieved by minimizing the contraction factor $\alpha$ under certain input and output constraints. On the input side, there is a chance constraint, that is, the best-practice (minimum) cost must not exceed the observed cost more than $\gamma \%$ of the time. With regard to the output side, there are chance constraints that each observed output must not exceed its maximum by more than $\gamma \%$ of the time. Finally, there is a convexity constraint, that is, the sum of the intensity coefficients equals 1. $\gamma$ is interpreted as the tolerance level for the chance constraint.

In stochastic efficiency program (9), the random inputs and outputs of firm $j$ are expressed as follows:

10 $${\widetilde{x}}_{ij}={x}_{ij}+{a}_{ij}{\varepsilon }_{ij} \left(\forall i\right), \mathrm{and} {\widetilde{y}}_{rj}={y}_{rj}+{b}_{rj}{\eta }_{rj} \left(\forall r\right),$$

where the error terms ${a}_{ij}{\varepsilon }_{ij} \mathrm{and} {b}_{rj}{\eta }_{rj}$ represent the symmetric disturbances of inputs and outputs, respectively, which arise because of factors that are entirely outside the firm’s control. Furthermore, ${\varepsilon }_{ij}$ $\left(\forall i\right)$ and ${\eta }_{rj}$ $\left(\forall r\right)$ are all assumed to follow a multivariate normal distribution, with zero means, that is, $E({\varepsilon }_{ij})=E({\eta }_{rj})=0 \left(\forall i,r\right)$, and unity variances, that is, $Var({\varepsilon }_{ij})=Var({\eta }_{rj})=1 \left(\forall i,r\right)$. Then, for each firm $j (j=1,\dots ,n)$, ${\widetilde{x}}_{ij}\sim N\left({x}_{ij},{a}_{ij}^{2}\right)$ and ${\widetilde{y}}_{rj}\sim N\left({y}_{rj},{b}_{rj}^{2}\right)$, where ${E({\widetilde{x}}_{ij})=x}_{ij}$ $\left(\forall i\right)$, ${E({\widetilde{y}}_{rj})=y}_{ij}$ $\left(\forall r\right)$, $Var({\widetilde{x}}_{ij})={a}_{ij}^{2}$ $\left(\forall i\right)$, and $Var\left({\widetilde{y}}_{rj}\right)={b}_{rj}^{2}$ $\left(\forall r\right)$.

The firm-specific symmetric disturbance terms—${\varepsilon }_{ij}$ $\left(\forall i\right)$ and ${\eta }_{rj}$ $\left(\forall r\right)$ allow the technology set and its resulting efficiency frontier to vary arbitrarily across firms and capture the presence of measurement and specification errors, if any, in the data. It is also important to note that these errors vary with the confidence level specified by the user.

Using the CC-programming theory, Land et al. (1993, 1994) proposed a method to transform the stochastic efficiency program (9) into a nonlinear programming (NLP) problem. However, solving this NLP problem requires information on a substantial number of parameters in the covariance matrix between the input and output components. Therefore, to reduce the computational time involved in the estimation of such parameters, Cooper et al. (1996) suggested a linearization method using a simplified assumption that the components of the inputs and outputs are related only through a common relationship with a single factor, which has long been used in economics (Sharpe 1963; Kahane 1977; Huang and Li 1996, 2001; Li 1995a, b, 1998; Li and Huang 1996. Following Cooper et al. (1996), we use this single-factor assumption in our proposed stochastic approach. In other words, ${\widetilde{x}}_{ij}={x}_{ij}+{a}_{ij}\varepsilon \left(\forall i\right)$ and ${\widetilde{y}}_{rj}={y}_{rj}+{b}_{rj}\varepsilon \left(\forall r\right)$ where $\varepsilon$ is a standard normal random variable with mean $E\left(\varepsilon \right)=0$ and a constant (finite) standard deviation $\sigma (\varepsilon )$.

The use of the single-factor assumption for linearization is not free. This simplifying assumption, as Cooper et al. (1996) rightly pointed out, requires further assumptions in the resulting linear program that the correlation coefficient between any two stochastic inputs/outputs, and between input and output, for any firm $j$ is always 1, that is, $\rho \left({\widetilde{{\varvec{x}}}}_{pj}, {\widetilde{{\varvec{x}}}}_{qj}\right)=1$ ($p\ne q)$, $\rho \left({\widetilde{{\varvec{y}}}}_{rj}, {\widetilde{{\varvec{y}}}}_{sj}\right)=1$ ($r\ne s)$, and $\rho \left({\widetilde{{\varvec{x}}}}_{pj}, {\widetilde{{\varvec{y}}}}_{sj}\right)=1$, which may not be easy to meet in practical applications. However, we believe that this convenient unit correlation coefficient assumption is important as it helps convert an NLP problem into an LP problem, which not only removes the inconveniences associated with solving an NLP problem but also helps one, as we will show later, compute the $SE$ behavior of firms using its dual.

Using the single factor assumption, we express, for each firm *j* ($j=1,\dots ,n)$, the constraint (9.1) as follows:

$$\begin{aligned} \tilde{h} = & \mathop \sum \limits_{j = 1}^{n} \lambda_{j} \left( {\mathop \sum \limits_{i = 1}^{m} w_{io} \tilde{x}_{ij} } \right) - \alpha \mathop \sum \limits_{i = 1}^{m} w_{io} \tilde{x}_{io} \\ = & \mathop \sum \limits_{j = 1}^{n} \lambda_{j} \left( {\mathop \sum \limits_{i = 1}^{m} w_{io} \left( {x_{ij} + a_{ij} {\tilde{\varepsilon }}} \right) } \right) - \alpha \mathop \sum \limits_{i = 1}^{m} w_{io} \left( {x_{io} + a_{io} {\tilde{\varepsilon }}} \right) \\ = & \mathop \sum \limits_{j = 1}^{n} \lambda_{j} \left( {\mathop \sum \limits_{i = 1}^{m} w_{io} x_{ij} } \right) + \mathop \sum \limits_{j = 1}^{n} \lambda_{j} \left( {\mathop \sum \limits_{i = 1}^{m} w_{io} a_{ij} {\tilde{\varepsilon }}} \right) - \alpha \mathop \sum \limits_{i = 1}^{m} w_{io} x_{io} - \alpha \mathop \sum \limits_{i = 1}^{m} w_{io} a_{io} {\tilde{\varepsilon }} \\ \end{aligned}$$

Hence, we can see that $\widetilde{h}\sim N\left(\sum_{j=1}^{n}{\lambda }_{j}\left(\sum_{i=1}^{m}{w}_{io}{x}_{ij}\right)-\alpha \sum_{i=1}^{m}{w}_{io}{x}_{io}, {\left(\sum_{j=1}^{n}{\lambda }_{j}\left(\sum_{i=1}^{m}{w}_{io}{a}_{ij}\right)-\alpha \sum_{i=1}^{m}{w}_{io}{a}_{io}\right)}^{2}\right) .$

Now, consider a new variable $\widetilde{z}=\frac{\widetilde{h}-E(\widetilde{h})}{{\sigma }_{\widetilde{h}}}$ that follows a standard normal distribution. As per the Central Limit Theorem, we have ${\mathbb{P}}\left(\widetilde{z}\le \frac{-\left(\sum_{j=1}^{n}{\lambda }_{j}\left(\sum_{i=1}^{m}{w}_{io}{x}_{ij}\right)-\alpha \sum_{i=1}^{m}{w}_{io}{x}_{io}\right)}{\tilde{\sigma }\left|\sum_{j=1}^{n}{\lambda }_{j}\left(\sum_{i=1}^{m}{w}_{io}{a}_{ij}\right)-\alpha \sum_{i=1}^{m}{w}_{io}{a}_{io}\right|}\right)\ge 1-\gamma .$ Then, $\Phi \left(\frac{-\left(\sum_{j=1}^{n}{\lambda }_{j}\left(\sum_{i=1}^{m}{w}_{io}{x}_{ij}\right)-\alpha \sum_{i=1}^{m}{w}_{io}{x}_{io}\right)}{\tilde{\sigma }\left|\sum_{j=1}^{n}{\lambda }_{j}\left(\sum_{i=1}^{m}{w}_{io}{a}_{ij}\right)-\alpha \sum_{i=1}^{m}{w}_{io}{a}_{io}\right|}\right)\ge 1-\gamma .$ Therefore, using the property of invertibility of $\Phi (\bullet )$, we have $\frac{-\left(\sum_{j=1}^{n}{\lambda }_{j}\left(\sum_{i=1}^{m}{w}_{io}{x}_{ij}\right)-\alpha \sum_{i=1}^{m}{w}_{io}{x}_{io}\right)}{\tilde{\sigma }\left|\sum_{j=1}^{n}{\lambda }_{j}\left(\sum_{i=1}^{m}{w}_{io}{a}_{ij}\right)-\alpha \sum_{i=1}^{m}{w}_{io}{a}_{io}\right|}\ge {\Phi }^{-1}\left(1-\gamma \right).$

Consequently, the deterministic form of Constraint (9.1) is expressed as $\sum_{j=1}^{n}{\lambda }_{j}\left(\sum_{i=1}^{m}{w}_{io}{x}_{ij}\right)-{\Phi }^{-1}(\gamma )\left|\sum_{j=1}^{n}{\lambda }_{j}\left(\sum_{i=1}^{m}{w}_{io}{a}_{ij}\right)-\alpha \sum_{i=1}^{m}{w}_{io}{a}_{io}\right|\le \alpha \sum_{i=1}^{m}{w}_{io}{x}_{io}.$ Similarly, Constraint (9.2) can be expressed as $\sum_{j=1}^{n}{\lambda }_{j} {y}_{rj}+{\Phi }^{-1}\left(\gamma \right)\tilde{\sigma } \left|\sum_{j=1}^{n}{\lambda }_{j} {b}_{rj}-\beta {b}_{ro}\right|\ge \beta {y}_{ro} (\forall r).$

Thus, we set up the deterministic version of program (9) as

11 $$\overline{\alpha }\left(\beta \right)=\mathrm{min}\alpha$$

$$s.t.\sum_{j=1}^{n}{\lambda }_{j}\left(\sum_{i=1}^{m}{w}_{io}{x}_{ij}\right)-{\Phi }^{-1}\left(\gamma \right)\left|\sum_{j=1}^{n}{\lambda }_{j}\left(\sum_{i=1}^{m}{w}_{io}{a}_{ij}\right)-\alpha \sum_{i=1}^{m}{w}_{io}{a}_{io}\right|\le \alpha \sum_{i=1}^{m}{w}_{io}{x}_{io},$$

$$\sum_{j=1}^{n}{\lambda }_{j} {y}_{rj}+{\Phi }^{-1}\left(\gamma \right)\overline{\sigma }\left|\sum_{j=1}^{n}{\lambda }_{j} {b}_{rj}-\beta {b}_{ro}\right|\ge \beta {y}_{ro} (\forall r),$$

$$\sum_{j=1}^{n}{\lambda }_{j}=1,$$

$${\lambda }_{j}\ge 0 \left(\forall j\right),$$ and $$\alpha :\mathrm{free}.$$

Program (11) is nonlinear owing to the existence of an absolute function. Using the goal programming theory of Charnes and Cooper (1977), we transformed it into a quadratic programming problem.^7^ To proceed, we use the following transformations:

$$\left|\sum_{j=1}^{n}{\lambda }_{j}\left(\sum_{i=1}^{m}{w}_{io}{a}_{ij}\right)-\alpha \sum_{i=1}^{m}{w}_{io}{a}_{io}\right|={p}^{+}+{p}^{-},$$

$$\sum_{j=1}^{n}{\lambda }_{j}\left(\sum_{i=1}^{m}{w}_{io}{a}_{ij}\right)-\alpha \sum_{i=1}^{m}{w}_{io}{a}_{io}={p}^{+}-{p}^{-},$$

$${p}^{+}{p}^{-}=0,$$

$$\left|\sum_{j=1}^{n}{\lambda }_{j} {b}_{rj}-\beta {b}_{ro}\right|={q}_{r}^{+}+{q}_{r}^{-} (\forall r),$$

$$\sum_{j=1}^{n}{\lambda }_{j} {b}_{rj}-\beta {b}_{ro}={q}_{r}^{+}-{q}_{r}^{-} (\forall r),$$

$${q}_{r}^{+}{q}_{r}^{-}=0 \left(\forall r\right).$$

The existence of two constraints, − $${p}^{+}{p}^{-}=0 \mathrm{and } {q}_{r}^{+}{q}_{r}^{-}=0$$, in our suggested transformations makes program (11) non-linear. However, if an LP problem exhibits an optimal solution vector, it also has an extreme optimal solution vector in which at least one of the variables from ($${p}^{+}, {p}^{-})$$ and $$({q}_{r}^{+}, {q}_{r}^{-})$$ is zero. Hence, the nonlinear constraints $${p}^{+}{p}^{-}=0$$ and $${q}_{r}^{+}{q}_{r}^{-}=0$$ can be safely ignored. Consequently, using the simplex algorithm, program (11) can be solved to find extreme optimal solutions.

Using the above transformations and notations, program (11) is converted into the following:

12 $$\overline{\alpha }\left(\beta \right)=\mathrm{min}\alpha$$

$$s.t$$.$$\sum_{j=1}^{n}{\lambda }_{j}\left(\sum_{i=1}^{m}{w}_{io}{x}_{ij}\right)-{\Phi }^{-1}\left(\gamma \right)\tilde{\sigma }\left({p}^{+}+{p}^{-}\right)\le \alpha \sum_{i=1}^{m}{w}_{io}{x}_{io},$$

$$\sum_{j=1}^{n}{\lambda }_{j}\left(\sum_{i=1}^{m}{w}_{io}{a}_{ij}\right)-\alpha \sum_{i=1}^{m}{w}_{io}{a}_{io}={p}^{+}-{p}^{-},$$

$$\sum_{j=1}^{n}{\lambda }_{j} {y}_{rj}+{\Phi }^{-1}\left(\gamma \right)\left({q}_{r}^{+}+{q}_{r}^{-}\right)\ge \beta {y}_{ro} (\forall r),$$

$$\sum_{j=1}^{n}{\lambda }_{j} {b}_{rj}-\beta {b}_{ro}={q}_{r}^{+}-{q}_{r}^{-} (\forall r),$$

$$\sum_{j=1}^{n}{\lambda }_{j}=1,$$

$$\lambda ,{p}^{+},{p}^{-},{q}^{+},{q}^{-}\ge 0,$$ and $$\alpha :\mathrm{free}.$$

### Stochastic value-based ${\varvec{S}}{\varvec{E}}$ measure

To compute the value-based measure of $SE$ of firm $o$, we set up the dual program (12) as

13 $$\overline{\alpha }\left(\beta \right)=\mathrm{max }\beta \left(\sum_{r=1}^{S}({u}_{r}{y}_{ro}+{\mu }_{r}{b}_{ro})\right)+{\omega }_{0}$$

$$s.t. \sum_{r=1}^{S}\left({u}_{r}{y}_{rj}+{\mu }_{r}{b}_{rj}\right)-\sum_{i=1}^{m}{w}_{io}\left(v{x}_{ij}-\vartheta {a}_{ij}\right)+{\omega }_{0}\le 0 \left(\forall j\right),$$

$$\sum_{i=1}^{m}{w}_{io}\left(v{x}_{io}-\vartheta {a}_{io}\right)=1,$$

$${\Phi }^{-1}\left(\gamma \right)v-\vartheta \le 0,$$

$${\Phi }^{-1}\left(\gamma \right)v+\vartheta \le 0,$$

$${\Phi }^{-1}\left(\gamma \right)\sum_{r=1}^{S}{u}_{r}-\sum_{r=1}^{S}{\mu }_{r}\le 0,$$

$${\Phi }^{-1}\left(\gamma \right)\sum_{r=1}^{S}{u}_{r}+\sum_{r=1}^{S}{\mu }_{r}\le 0,$$

$$v,u\ge 0, \vartheta ,\mu ,{\omega }_{0}: \mathrm{free}.$$

For each firm $o\in \left\{1,\dots ,n\right\}$, its transformation function is considered as follows:

14 $$\psi \left(\overline{\alpha }\left(\beta \right){x}_{o},\overline{\alpha }\left(\beta \right){a}_{o},\beta {y}_{o},\beta {b}_{o}\right)\equiv \beta \sum_{r=1}^{S}\left({u}_{r}{y}_{ro}+{\mu }_{r}{b}_{ro}\right)-\overline{\alpha }\left(\beta \right)\sum_{i=1}^{m}{w}_{io}\left(v{x}_{io}-\vartheta {a}_{io}\right)+{\omega }_{0}=0$$

On differentiation of (14) with respect to $\beta$ yields

15 $$\begin{aligned} \frac{\partial \psi \left( \cdot \right)}{{\partial \beta }} & = \mathop \sum \limits_{r = 1}^{s} \frac{\partial \psi \left( . \right)}{{\partial \left( {\beta y_{ro} } \right)}}y_{ro} + \mathop \sum \limits_{r = 1}^{s} \frac{\partial \psi \left( \cdot \right)}{{\partial \left( {\beta b_{ro} } \right)}}b_{ro} + \mathop \sum \limits_{i = 1}^{m} \frac{\partial \psi \left( . \right)}{{\partial \left( {\alpha x_{io} } \right)}}x_{io} \frac{\partial \alpha }{{\partial \beta }} + \mathop \sum \limits_{i = 1}^{m} \frac{\partial \psi \left( . \right)}{{\partial \left( {\alpha a_{io} } \right)}}a_{io} \frac{\partial \alpha }{{\partial \beta }} = 0, \\ \Rightarrow \frac{\partial \alpha }{{\partial \beta }} & = - \frac{{\mathop \sum \nolimits_{r = 1}^{s} \frac{\partial \psi \left( \cdot \right)}{{\partial \left( {\beta y_{ro} } \right)}}y_{ro} + \mathop \sum \nolimits_{r = 1}^{s} \frac{\partial \psi \left( \cdot \right)}{{\partial \left( {\beta b_{ro} } \right)}}b_{ro} }}{{\mathop \sum \nolimits_{i = 1}^{m} \frac{\partial \psi \left( \cdot \right)}{{\partial \left( {\alpha x_{io} } \right)}}x_{io} + \mathop \sum \nolimits_{i = 1}^{m} \frac{\partial \psi \left( \cdot \right)}{{\partial \left( {\alpha a_{io} } \right)}}a_{io} }} \\ & = - \frac{{\mathop \sum \nolimits_{r = 1}^{s} u_{r} y_{ro} + \mathop \sum \nolimits_{r = 1}^{s} \mu_{r} b_{ro} }}{{ - \mathop \sum \nolimits_{i = 1}^{m} vw_{io} x_{io} + \mathop \sum \nolimits_{i = 1}^{m} \vartheta w_{io} a_{io} }} \\ & = \frac{{\overline{\alpha }\left( \beta \right)\mathop \sum \nolimits_{i = 1}^{m} w_{io} \left( {vx_{io} - \vartheta a_{io} } \right) - \omega_{0} }}{\beta } = \frac{{\overline{\alpha }\left( \beta \right) - \omega_{0} }}{\beta } \\ \end{aligned}$$

The (input-oriented) value-based measure of $SE$ of firm $o$ can then be obtained as follows:

16 $$\varepsilon \left(\gamma \right)=\frac{\partial \overline{\alpha }(\beta )}{\partial \beta } \frac{\beta }{\overline{\alpha }(\beta )}=\frac{\overline{\alpha }\left(\beta \right)-{\omega }_{0}}{\beta }\frac{\beta }{\overline{\alpha }(\beta )}=\frac{\overline{\alpha }\left(\beta \right)-{\omega }_{0}}{\overline{\alpha }(\beta )}$$

Because DEA technologies are not smooth at vertices, we face multiple optimal values for ${\omega }_{0}$. Therefore, to compute the maximum (minimum) value of ${\omega }_{0}$ for firm $o$, we set the following program.

17 $${\omega }_{0}^{+}\left({\omega }_{0}^{-}\right)=\mathrm{max} (\mathrm{min}) {\omega }_{0}$$

$$s.t.\beta \left(\sum_{r=1}^{S}({u}_{r}{y}_{ro}+{\mu }_{r}{b}_{ro})\right)+{\omega }_{0}=\overline{\alpha }\left(\beta \right),$$

$$\sum_{r=1}^{S}\left({u}_{r}{y}_{rj}+{\mu }_{r}{b}_{rj}\right)-\sum_{i=1}^{m}{w}_{io}\left(v{x}_{ij}-\vartheta {a}_{ij}\right)+{\omega }_{0}\le 0, (\forall j\ne o)$$

$$\sum_{i=1}^{m}{w}_{io}\left(v{x}_{io}-\vartheta {a}_{io}\right)=1,$$

$${\Phi }^{-1}\left(\gamma \right)v-\vartheta \le 0,$$

$${\Phi }^{-1}\left(\gamma \right)v+\vartheta \le 0,$$

$${\Phi }^{-1}\left(\gamma \right)\sum_{r=1}^{S}{u}_{r}-\sum_{r=1}^{S}{\mu }_{r}\le 0,$$

$${\Phi }^{-1}\left(\gamma \right)\sum_{r=1}^{S}{u}_{r}+\sum_{r=1}^{S}{\mu }_{r}\le 0,$$

$$v,u\ge 0,\, \vartheta ,\mu ,{\omega }_{0}: \mathrm{free}.$$

Using the optimal values of program (17), the (input-oriented) right- and left-hand value-based $SE$ scores of firm $o$ can be calculated as follows:

18 $${\varepsilon }^{+}\left(\gamma \right)=\frac{\overline{\alpha }\left(\beta \right)-{\omega }_{0}^{-}}{\overline{\alpha }\left(\beta \right)},\mathrm{ and}\, {\varepsilon }^{-}\left(\gamma \right)=\frac{\overline{\alpha }\left(\beta \right)-{\omega }_{0}^{+}}{\overline{\alpha }\left(\beta \right)}.$$

We present Theorem 1 to determine, using the formulae in (18), the returns-to-scale types at each confidence level given the values of ${\omega }_{0}^{-}$ and ${\omega }_{0}^{+}$.

#### Theorem 1

For every confidence level $\gamma$, the (input-oriented) returns to scale of firm $o$ are (a) decreasing (DRS) (i.e., ${\varepsilon }_{I}^{-}\left(\gamma \right)>1$) if ${\omega }_{0}^{+}<0,$ (b) constant (CRS) (i.e., ${\varepsilon }_{I}^{-}\left(\gamma \right)\le 1\le {\varepsilon }_{I}^{+}\left(\gamma \right))$ if ${\omega }_{0}^{-}\le 0\le {\omega }_{0}^{+},$ and (c) increasing (IRS) (i.e., ${\varepsilon }_{I}^{+}\left(\gamma \right)<1$) if ${\omega }_{0}^{-}>0.$

#### Proof

Let ${v}^{*},{u}^{*}, {\vartheta }^{*},{\mu }^{*}$, and ${\omega }_{0}^{*}$ be optimal solutions of the LP program (13) for the radial efficient firm $({\widetilde{x}}_{o}, {\widetilde{y}}_{o})$, which is defined according to (10) as ${\widetilde{x}}_{io}={x}_{io}+{a}_{io}{\varepsilon }_{io} (\forall i)$ and ${\widetilde{y}}_{ro}={y}_{ro}+{b}_{ro}{\eta }_{ro} \left(\forall r\right).$ So, $\sum_{r=1}^{S}\left({u}_{r}^{*}{y}_{ro}+{\mu }_{r}^{*}{b}_{ro}\right)+{\omega }_{0}^{*}=\sum_{i=1}^{m}{w}_{io}\left({v}^{*}{x}_{io}-{\vartheta }^{*}{a}_{io}\right)=1$. Furthermore, $\sum_{r=1}^{S}\left({u}_{r}^{*}{y}_{rj}+{\mu }_{r}^{*}{b}_{rj}\right)-\sum_{i=1}^{m}{w}_{io}\left({v}^{*}{x}_{ij}-{\vartheta }^{*}{a}_{ij}\right)+{\omega }_{0}^{*}=0$ is a supporting hyperplane to ${T}^{V(S)}$ in (7), and the corresponding value at $\left(\overline{\alpha }\left(\beta \right), \beta \right)$ is presented in (14). Differentiating (14) for $\beta$, the following measure of SE was used: $\varepsilon \left(\gamma \right)=\frac{\overline{\alpha }\left(\beta \right)-{\omega }_{o}^{*}}{\overline{\alpha }\left(\beta \right)}$. The right- and left-hand SEs can then be easily computed as ${\varepsilon }^{+}\left(\gamma \right)=\frac{\overline{\alpha }\left(\beta \right)-{\omega }_{o}^{*-}}{\overline{\alpha }\left(\beta \right)} \mathrm{and} {\varepsilon }^{-}\left(\gamma \right)=\frac{\overline{\alpha }\left(\beta \right)-{\omega }_{o}^{*+}}{\overline{\alpha }\left(\beta \right)}$, respectively. It then immediately follows that

- ${\varepsilon }^{-}\left(\gamma \right)>1$if ${\omega }_{o}^{*+}<0$ and since ${\omega }_{o}^{*-}<{\omega }_{o}^{*+}$, then ${\varepsilon }^{+}\left(\gamma \right)>1$. Thus, returns to scale decrease (DRS).
- If ${\omega }_{o}^{*-}<0$ then ${\varepsilon }^{+}\left(\gamma \right)>1$ and if ${\omega }_{o}^{*+}>0$ then ${\varepsilon }^{-}\left(\gamma \right)<1$. Therefore, ${\varepsilon }^{-}\left(\gamma \right)\le 1\le {\varepsilon }^{+}\left(\gamma \right)$ and returns to scale are constant (CRS).
- If ${\omega }_{o}^{*-}>0$ then ${\varepsilon }^{+}\left(\gamma \right)<1$ and since ${\omega }_{o}^{*+}>{\omega }_{o}^{*-}$ then ${\varepsilon }^{-}\left(\gamma \right)<1$. Thus, returns to scale (IRS) are increasing.
  $$\square$$

Regarding the returns-to-scale types between deterministic and stochastic cases, we present Theorem 2.

#### Theorem 2

For a predetermined significance level of $\gamma =0.5$, the returns-to-scale types obtained from deterministic and stochastic programs were the same.

#### Proof

To prove this, it suffices to show that for $\gamma =0.5$, programs (12) and (3) are equivalent. To this end, since programs (12) and (11) are equivalent and ${\Phi }^{-1}\left(0.5\right)=0$, replacing it in the constraints of program (11) yields program (3).

$$\square$$

For the visual presentation of technology, we consider in Table 1 a hypothetical dataset of six firms, each producing one output using one input.

**Table 1:** Hypothetical data set

Firm	Input: $x (a,b)$	Output: $y (a,b)$
A	(1.5, 0.5)	(1, 0.51)
B	(4, 0.4)	(6, 0.6)
C	(6, 0.6)	(7, 0.7)
D	(2, 0.2)	(2.5, 2.5)
E	(4, 0.4)	(3.5, 0.5)
F	(7, 0.7)	(6.5, 0.1)

For $(a,b)$, $a$ is the mean and $b$ is the standard deviation

**Fig. 2:**
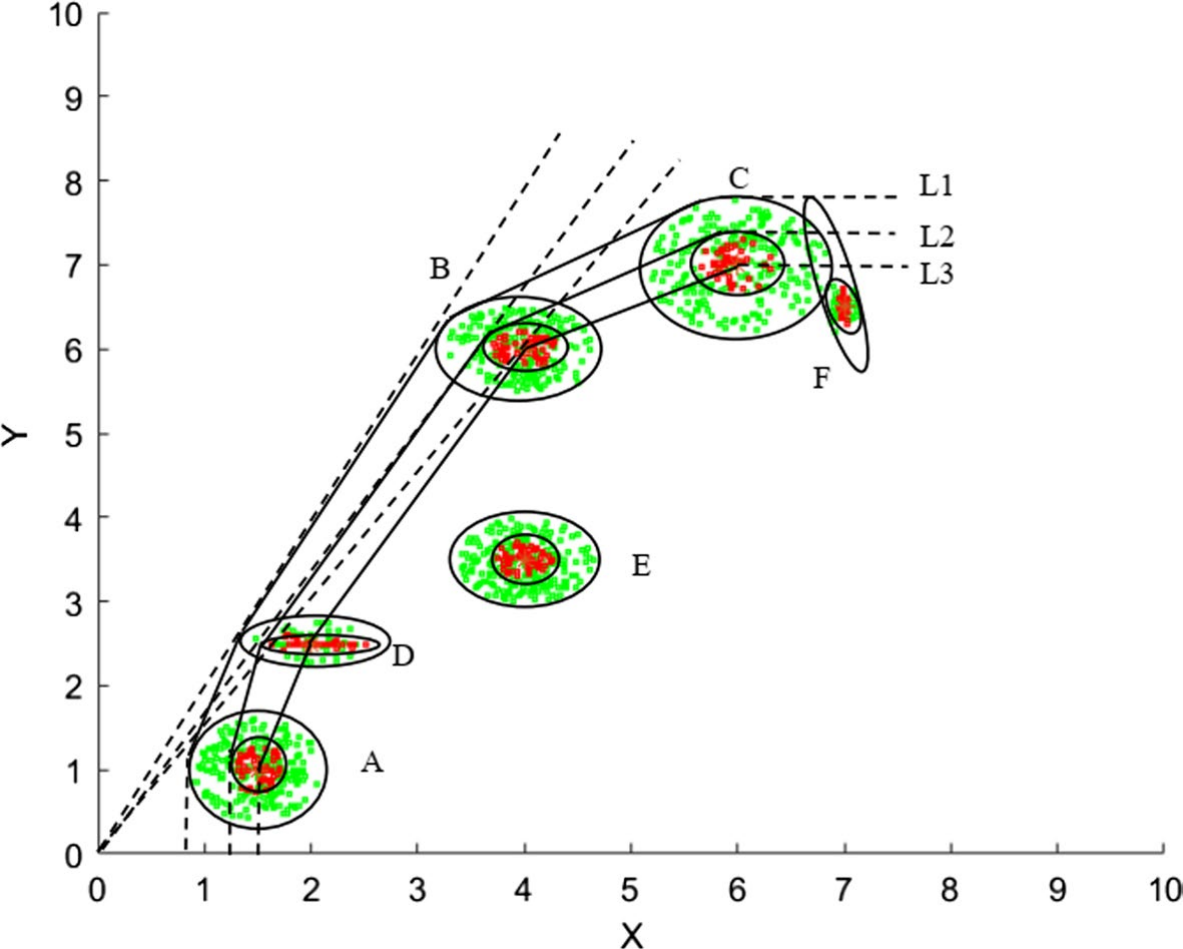
DEA stochastic production frontiers under chance constraints at $\gamma =0.5, 0.3$ and $0.1$

Based on the data in Table 1, we constructed the stochastic production frontiers in Fig. 2 by considering three different levels of tolerance ($\gamma =0.1, 0.3,\mathrm{ and }0.5$).^8^

The piecewise linear line (L3) passing through the mean points of the centers of circles represents the production frontier at the tolerance level of 0.5 and according to Theorem 2, this stochastic frontier is the same as the deterministic frontier. Similarly, lines L2 and L1 represent the production frontiers at tolerance levels $\mathrm{of} 0.3$ and 0.1, respectively. The TE scores at these tolerance levels are presented in Table 2. At each of these three tolerance levels, four firms, A, B, C, and D, are consistently found to be technically efficient. Of the remaining two firms, F becomes efficient at a tolerance level of 0.1, but inefficient at tolerance levels of $0.3$ and 0.5. However, E was inefficient at all three tolerance levels. This finding suggests that a firm’s efficiency status in a deterministic environment can be subject to change in the stochastic case.

**Table 2:** Stochastic TE and SE scores of six firms

Firm	$\gamma =0.1$				$\gamma =0.3$				$\gamma =0.5$			
	$T{E}_{o}$	${\varepsilon }_{1}^{-}$	${\varepsilon }_{1}^{+}$	$\mathrm{RTS}$	$T{E}_{o}$	${\varepsilon }_{1}^{-}$	${\varepsilon }_{1}^{+}$	$\mathrm{RTS}$	$T{E}_{o}$	${\varepsilon }_{1}^{-}$	${\varepsilon }_{1}^{+}$	$\mathrm{RTS}$
$\mathrm{A}$	1	0	0.6400	IRS	1	2.220e−16	0.3900	IRS	1	0	0.2230	IRS
$\mathrm{B}$	1	0.5630	3	CRS	1	0.6680	3	CRS	1	0.8570	3	CRS
$\mathrm{C}$	1	2.3340	$\infty$	DRS	1	2.3340	$\infty$	DRS	1	2.3340	$\infty$	DRS
$\mathrm{D}$	1	0.0720	5.2010	CRS	1	0.2440	1.5230	CRS	1	0.4170	0.7140	IRS
$\mathrm{E}$	0.7450	0.4140	0.4140	IRS	0.713	0.5340	0.5340	IRS	0.642	0.7780	0.7780	IRS
$\mathrm{F}$	1	3.3850	$\infty$	DRS	0.801	2.4270	2.4270	DRS	0.714	2.6000	2.6000	DRS

*RTS* returns to scale

Input-oriented right- and left-hand SE scores were computed using value-based technology (7) in a stochastic environment $({T}^{V(S)})$, the input-oriented right- and left-hand $SE$ scores are computed. Table 2 presents the results.

At each of these three tolerance levels considered, out of the four stochastically technically efficient firms (A, B, C, and D), B is CCR-efficient and hence exhibits CRS because its left-hand SE scores are all less than 1, and the right-hand SE scores are all greater than 1. D is CCR-efficient only at tolerance levels of $0.1$ and 0.3 and hence exhibits CRS at those tolerance levels, but IRS at the tolerance level of 0.5 (as its right-hand SE score is less than 1). Of the remaining two stochastically technically efficient firms, A exhibits IRS (as its right-hand SE score is less than 1) and C exhibits DRS (as its left-hand SE score is greater than 1) at each of the three tolerance levels. Finally, regarding the returns-to-scale types of inefficient firms, since E’s projection onto the frontier is located on the interior point of the facet DB (see Fig. 2), its right- and left-hand SE values are all equal, but all less than one, implying IRS. As is evident from Table 2, the efficiency status of F is the mix, that is, it is efficient at a tolerance level of 0.1, but inefficient at other tolerance levels. However, at all these tolerance levels, DRS is exhibited because its left-hand SE values are all greater than one. The finding that D exhibits IRS at the tolerance level of 0.5, but CRS at the other tolerance levels, indicates that the returns-to-scale characterizations of firms in a deterministic technology is subject to change in the stochastic case.

## The illustrative empirical application

We now demonstrate an illustrative empirical application of our proposed stochastic efficiency program using a dataset used earlier by Sahoo and Tone (2009a, b) for analyzing capacity utilization and profit change behaviors of Indian banks for eight years (1998–2005).

### The data

The dataset consists of 71 banks (26 public banks, 27 private banks, and 18 foreign banks), each using three inputs, borrowed funds $\left({x}_{1}\right)$, fixed assets $\left({x}_{2}\right)$, and labor $\left({x}_{3}\right)$, to produce three outputs, investments $\left({y}_{1}\right)$, performing loan assets $\left({y}_{2}\right)$, and non-interest income $\left({y}_{3}\right)$. All monetary values of the input and output quantities are deflated by the wholesale price index with a base of 1993–94 to obtain their present (implicit) quantities. All monetary values are measured for Indian rupees (Rs.) for crores (Rs. 1 crore = Rs. 10 million). Concerning the prices of inputs, the unit prices of ${x}_{1}$, ${x}_{2}$, and ${x}_{3}$ are measured as the average interest paid per rupee of ${x}_{1}\left({w}_{1}\right)$, non-labor operational cost per rupee amount of ${x}_{2}$ $\left({w}_{2}\right)$, and average staff cost $\left({w}_{3}\right)$, respectively. The input and output quantities are all assumed to be random, with each following a normal distribution with a known mean (presented in Table 3) and known standard deviation (shown in Table 4).

**Table 3:** The means of the inputs and outputs along with unit input prices of 71 banks

Banks	${x}_{1}$	${x}_{2}$	${x}_{3}$	${w}_{1}$	${w}_{2}$	${w}_{3}$	${y}_{1}$	${y}_{2}$	${y}_{3}$
1	68.1000	14.6454	231,072.8140	1.5596	0.9909	0.000177	768.8273	1736.3488	24.8453
2	0.4325	0.4983	20,422.9728	31.4076	1.2377	0.000144	29.9458	75.3437	1.3874
3	0.6667	0.5792	13,155.2839	21.4205	1.3339	0.000170	51.8814	113.9078	1.6282
4	1.2824	0.2103	28,744.5519	10.7181	1.9953	0.000191	20.9039	57.0878	0.9490
5	0.3710	0.5541	13,069.0440	180.5710	1.0925	0.000150	23.8559	73.6597	0.9581
6	0.9593	0.2079	6239.0567	16.4462	2.6319	0.000193	18.2914	45.7601	0.6654
7	0.4479	0.4409	11,555.5328	30.8446	1.2367	0.000156	32.1166	82.5182	1.1282
8	1.0401	0.2961	2915.5739	3.5463	1.5125	0.000378	28.8480	45.7574	0.9235
9	12.6481	4.1937	39,585.8189	4.6379	0.6836	0.000158	116.3621	388.2644	4.3501
10	0.8532	2.3304	21,087.5006	14.2102	0.4688	0.000154	52.0073	127.5979	1.4490
11	12.0941	4.7308	44,548.3420	1.9544	0.5471	0.000227	110.0780	347.2673	5.0368
12	1.5552	0.7480	14,290.2602	4.5448	0.9576	0.000213	51.6128	97.7401	1.2481
13	8.1571	4.0783	47,897.6398	3.1790	0.6338	0.000196	127.8455	353.1985	5.3079
14	1.9884	1.7729	13,814.3647	7.0141	0.5274	0.000235	38.4213	107.2447	1.1359
15	1.5104	1.8159	25,543.8390	22.5708	0.7228	0.000166	70.5957	177.8313	1.8718
16	2.1951	4.7242	46,825.7087	9.4810	0.4216	0.000152	117.4315	250.4290	2.8011
17	1.3604	4.9417	28,271.2984	13.5322	0.3458	0.000174	86.9031	216.1909	1.8353
18	3.3363	4.5442	58,524.2537	7.6916	0.5729	0.000167	150.5229	367.8565	4.6353
19	1.0626	1.1892	20,023.4103	8.4986	0.5132	0.000144	67.5708	111.9863	1.0442
20	1.9417	2.6937	27,801.8350	6.4161	0.3016	0.000153	64.9848	139.3250	1.7818
21	1.5121	1.8591	27,802.6741	9.8363	0.6378	0.000222	63.5813	170.5501	1.6023
22	1.2593	0.5976	12,837.2153	6.7137	1.3492	0.000189	58.6636	874.6757	1.0476
23	5.5382	0.8763	9963.8262	3.0145	0.9697	0.000122	41.1079	98.6003	1.5917
24	1.0343	0.7889	13,282.6539	22.1694	1.5211	0.000166	65.6004	149.2672	1.5780
25	0.7387	0.5508	10,322.7455	26.9226	1.0522	0.000208	33.4490	74.9515	0.9615
26	1.3739	1.0360	13,619.3051	4.8019	0.6399	0.000160	35.4865	79.1503	1.0087
27	0.5736	0.8526	1260.9627	9.7425	0.1872	0.000119	6.8275	22.2569	0.2634
28	1.8213	0.6552	631.8055	2.9297	0.6389	0.000122	13.3391	41.9503	0.6856
29	9.8062	3.0160	1338.4830	0.9666	0.3366	0.000192	65.5371	80.8818	1.1227
30	0.1499	0.5331	4283.9222	19.1395	0.3716	0.000088	10.3505	26.7194	0.3456
31	0.0271	0.0655	1301.1018	34.3294	0.7699	0.000025	3.9768	6.7571	0.1018
32	0.2747	0.1868	3151.8460	15.5240	0.5042	0.000103	6.2410	18.4547	0.2733
33	0.1712	0.1112	1667.3429	5.4753	0.4358	0.000082	3.8861	10.3796	0.1622
34	0.8751	0.6152	2687.5908	2.7939	1.0740	0.000377	6.9970	25.3106	0.8604
35	3.9433	2.2770	204.4933	2.4176	0.2471	0.019207	22.4117	67.1690	0.4804
36	4.8966	1.6228	729.0985	1.5649	0.1823	0.000586	36.2109	87.2141	0.2525
37	5.7535	1.9180	6033.0447	0.6133	0.1961	0.000031	9.5859	27.3903	0.7109
38	1.5520	0.7886	1130.7411	4.4120	0.1313	0.000041	7.9185	19.5764	0.1596
39	2.1426	0.8877	1098.0398	2.0347	0.4886	0.000787	18.7017	57.1618	0.3718
40	0.7647	0.4895	1271.5057	3.7382	0.1536	0.000430	17.5242	37.2371	0.1394
41	0.3138	0.1475	217.3686	2.1462	0.2528	0.000445	3.3779	8.9357	0.0053
42	20.3317	0.6996	3596.8108	9.5953	0.2743	0.000169	31.6193	58.6743	0.2887
43	4.5754	0.6681	2683.5745	0.5194	0.2737	0.000076	13.4838	30.2588	0.3493
44	0.1662	0.1690	1853.7080	6.9180	0.6453	0.000033	3.1125	11.8737	0.2173
45	0.0099	0.0581	955.6227	40.2915	1.0953	0.000019	0.2593	0.9470	0.0776
46	11.4310	3.4586	1621.1997	2.7468	0.0401	0.000135	38.4500	61.6207	0.0527
47	0.0786	0.1777	2143.8782	34.6337	0.9080	0.000077	4.2252	8.6918	0.3064
48	0.0442	0.0756	3847.0387	10.8397	1.8481	0.000014	1.3247	3.3803	0.5316
49	0.0163	0.2211	671.9332	43.9394	0.0937	0.000346	3.2050	9.0152	0.0131
50	0.2351	0.1958	3712.0727	11.1481	0.6135	0.000108	8.4520	19.7506	0.3476
51	0.0566	0.0253	794.3014	72.6109	2.9982	0.000071	1.7383	3.4084	0.1218
52	4.7373	1.9963	6372.9742	2.1178	0.1161	0.000098	16.0506	53.3586	0.3851
53	1.1853	0.7289	6040.1171	3.1510	0.1951	0.000072	10.5492	26.1635	0.3317
54	0.3488	0.0159	22.3365	0.1160	2.3726	0.000324	0.2847	0.7337	0.0050
55	12.5233	0.4258	332.5308	0.2781	2.5053	0.000160	13.2140	47.9448	1.2220
56	14.1786	0.2770	355.0173	0.1967	1.3702	0.000469	9.8742	35.1222	0.6311
57	0.5282	0.1357	309.5109	19.0387	2.8038	0.000311	1.9769	8.6026	0.4407
58	3.8787	0.6974	491.2626	0.4946	0.3861	0.000414	5.7095	14.6569	0.2208
59	1.7684	0.0102	33.1541	0.2008	4.9490	0.000501	0.5713	2.2969	0.0612
60	20.0390	3.0197	1585.4716	0.4730	1.0225	0.000517	35.4619	109.9327	3.8043
61	0.7479	0.0228	52.5203	1.1674	4.9703	0.000608	3.4626	3.6002	0.0643
62	2.7446	1.2555	79.2919	0.2268	0.2450	0.000987	3.1243	11.3549	0.1406
63	3.5145	0.0451	9156.4851	0.3727	2.1963	0.000005	9.6810	23.8449	0.1251
64	1.3650	0.0426	146.7803	0.4465	2.0252	0.000128	0.9449	3.4518	0.0483
65	0.8318	0.0166	90.2168	2.5030	3.3179	0.000780	2.5044	9.5684	0.1925
66	1.7777	0.0166	2341.9032	0.3176	4.9815	0.000005	1.0159	4.6030	0.0354
67	0.3953	0.1172	15.4043	5.1415	0.1803	0.000502	0.8731	2.6146	0.0364
68	0.4333	0.0106	204.2668	0.3773	2.7202	0.000481	0.3886	2.2331	0.0355
69	54.8725	1.2932	34.1071	0.4196	0.0338	0.023052	41.8377	177.0707	1.9755
70	22.5396	3.0623	2491.6622	3.3634	0.5975	0.000593	32.1198	74.2094	2.8352
71	2.7269	0.3002	80.5671	0.2399	0.2701	0.000721	1.3913	5.1017	0.0910

**Table 4:** The standard deviations of the inputs and outputs of 71 banks

Banks	${x}_{1}$	${x}_{2}$	${x}_{3}$	${y}_{1}$	${y}_{2}$	${y}_{3}$
1	10.2761	2.8507	9523.0570	361.1912	329.3894	3.3711
2	0.3243	0.1226	19,612.3100	7.3137	12.8256	0.2357
3	0.4355	0.0485	1566.2550	21.2321	38.1618	0.3244
4	1.1460	0.0098	35,723.0900	5.8070	12.7340	0.1739
5	0.4745	0.2443	116.0695	3.2547	6.1352	0.1533
6	0.7842	0.0121	1622.1800	5.5051	4.5941	0.0487
7	0.3802	0.1267	1129.0920	6.7264	13.4169	0.0973
8	0.5811	0.0879	1600.4690	21.1830	17.6628	0.3710
9	14.0536	0.1327	12,671.1500	16.4858	78.8113	0.4629
10	0.5370	0.1030	1759.8810	5.3334	17.7115	0.1709
11	3.7572	0.3490	9580.5270	14.3820	25.5055	0.8603
12	0.5947	0.0968	2498.8860	15.9466	24.6052	0.5078
13	2.8386	0.7122	13,017.6300	15.3522	61.1519	1.0284
14	1.7091	0.3821	1057.5330	5.1866	23.3821	0.1904
15	1.2107	0.1315	3475.0790	14.8787	22.5425	0.2866
16	1.1157	0.8577	3000.5590	18.8697	47.0327	0.2965
17	0.6380	0.5676	2961.7660	18.6649	33.1439	0.1841
18	1.1564	0.1176	8797.8970	29.6685	75.6047	0.5686
19	0.3051	0.1172	3327.3110	12.4341	14.9897	0.2111
20	1.0151	0.2984	4245.7350	8.5857	16.3334	0.5304
21	1.0645	0.2115	5150.4420	5.7536	33.1686	0.2882
22	0.2139	0.2652	2347.0150	28.1306	2146.9900	0.2542
23	5.4376	0.1951	366.1181	9.4957	21.9662	0.4251
24	1.0271	0.1328	1188.0290	18.3657	43.2375	0.5402
25	0.6978	0.0566	2219.1920	6.3389	12.7496	0.1524
26	0.7827	0.2157	2501.7380	7.5635	16.6562	0.4310
27	0.4717	0.1385	54.5427	2.1337	11.9903	0.1194
28	1.1364	0.2091	334.8054	2.9632	10.5197	0.2537
29	10.8137	2.3058	631.6041	65.0802	60.9556	0.5113
30	0.0936	0.0963	138.6136	2.7358	1.8060	0.0188
31	0.0178	0.0144	90.2233	1.5032	1.3241	0.0154
32	0.2187	0.0783	440.8488	1.4625	3.4097	0.0815
33	0.0720	0.0074	867.5313	1.3878	2.1066	0.0335
34	0.4870	0.4063	3281.2440	1.2412	7.7730	0.3566
35	2.8867	0.4770	279.2979	12.8282	33.2987	0.2372
36	3.0643	0.7197	320.2342	24.0317	81.2471	0.0504
37	3.7371	0.8729	355.3727	4.8044	13.3527	0.1038
38	1.2015	0.2860	204.2568	2.7486	10.0768	0.0596
39	0.6179	0.2039	534.8040	3.5879	11.2343	0.2256
40	0.0859	0.0376	88.1207	8.6354	9.6225	0.0332
41	0.0752	0.0740	37.5784	0.9857	1.8663	0.0014
42	34.0616	0.3700	957.1578	15.0030	20.9467	0.0752
43	2.8870	0.2829	1306.3720	6.7667	18.7212	0.0708
44	0.0820	0.0833	366.0991	0.4548	3.8405	0.0361
45	0.0105	0.0114	601.1055	0.1042	0.2297	0.0284
46	12.6386	4.3393	708.3910	40.2227	53.5951	0.0399
47	0.0595	0.1118	203.7611	1.9295	1.9406	0.0151
48	0.0306	0.0212	695.5238	0.4971	1.3035	0.1993
49	0.0067	0.0241	111.2063	0.2959	2.0643	0.0024
50	0.1560	0.0133	354.5148	3.2130	6.6751	0.1224
51	0.0920	0.0007	135.6133	0.8067	1.2338	0.0253
52	4.2837	0.2994	538.7854	1.2527	6.3336	0.1556
53	1.1213	0.1859	2816.5610	3.9267	6.4732	0.2708
54	0.1252	0.0089	5.3860	0.1415	0.2273	0.0025
55	5.3485	0.0688	283.1380	6.8033	28.1929	0.6568
56	2.6620	0.0941	250.3139	3.2598	19.4312	0.4657
57	0.9769	0.0383	15.3733	1.4458	4.3820	0.3090
58	2.2051	0.1250	225.0801	1.3696	4.4999	0.0511
59	1.5106	0.0024	1.7504	0.2358	1.2826	0.0409
60	14.5400	0.9226	223.5289	15.3532	30.8000	1.1175
61	0.6053	0.0176	4.7099	3.0749	1.0242	0.0104
62	0.8394	1.8307	6.6078	1.5231	6.2029	0.0916
63	2.2409	0.0118	1259.3690	12.4435	22.8410	0.0364
64	1.1995	0.0053	42.5258	0.1185	0.3645	0.0144
65	0.6396	0.0040	13.9585	0.6044	2.8504	0.0496
66	1.8802	0.0100	1231.6560	0.8970	3.5025	0.0127
67	0.3714	0.0188	16.0499	0.7412	2.1500	0.0122
68	0.2632	0.0020	476.7371	0.1782	1.3320	0.0350
69	62.4162	0.5402	18.7544	33.8122	243.9712	0.7001
70	20.7893	0.1909	2270.2630	12.4799	29.3829	1.8066
71	1.6991	0.0735	40.5675	0.6920	2.6638	0.0292

In DEA, if the number of DMUs ($n$) is less than the combined number of inputs (*m*) and outputs (*s*)*,* many DMUs are efficient. Therefore, $n$ must exceed ($m$ + $s$). The rule of thumb in the DEA literature suggests that $n$ ≥ *max* {$m$ × $s$*,* 3 × ($m$ +$s$)}. In our dataset, $n$ = 71, and $m$ = $s$  = 3. Therefore, the thumb rule was satisfied at 71 ≥ *max* {9, 18}.

We used the GAMS software on a machine with the following specifications to compute the efficiency and scale elasticity scores of banks: CPU, Intel Pentium 4 at 2 GHz and RAM, 1024 MB.

### Results

#### Stochastic value-based $TE$ scores

Table 5 presents the value-based TE scores of 71 banks in the stochastic environment at the three different confidence levels considered. As observed, only 38 banks were efficient at all three confidence levels. It is also interesting to observe that inefficient banks tend to experience a decline in their efficiency performance with an increase in $\gamma .$

**Table 5:** Stochastic value-based $TE$ scores of 71 banks

Banks	$\gamma =0.1$	$\gamma =0.3$	$\gamma =0.5$	Banks	$\gamma =0.1$	$\gamma =0.3$	$\gamma =0.5$
1	1.0000	1.0000	1.0000	37	0.1058	0.0969	0.0762
2	1.0000	1.0000	1.0000	38	0.0823	0.0823	0.0676
3	1.0000	1.0000	1.0000	39	1.0000	1.0000	1.0000
4	0.4174	0.4057	0.2934	40	1.0000	1.0000	0.9479
5	1.0000	1.0000	1.0000	41	0.7571	0.7507	0.7147
6	1.0000	1.0000	1.0000	42	1.0000	0.2072	0.0352
7	1.0000	1.0000	1.0000	43	0.1712	0.1641	0.1465
8	1.0000	1.0000	1.0000	44	1.0000	1.0000	1.0000
9	1.0000	1.0000	1.0000	45	1.0000	1.0000	1.0000
10	1.0000	1.0000	1.0000	46	0.4931	0.4785	0.4365
11	1.0000	1.0000	1.0000	47	1.0000	1.0000	1.0000
12	0.8750	0.7525	0.6702	48	1.0000	1.0000	1.0000
13	1.0000	1.0000	1.0000	49	1.0000	1.0000	1.0000
14	0.6154	0.5946	0.5046	50	0.5304	0.5207	0.5066
15	1.0000	1.0000	1.0000	51	1.0000	1.0000	0.4247
16	1.0000	1.0000	1.0000	52	1.0000	1.0000	1.0000
17	1.0000	1.0000	1.0000	53	0.2496	0.2264	0.1658
18	1.0000	1.0000	1.0000	54	1.0000	1.0000	1.0000
19	1.0000	1.0000	1.0000	55	0.9320	0.8328	0.7088
20	1.0000	1.0000	1.0000	56	0.6136	0.4690	0.3768
21	1.0000	1.0000	1.0000	57	1.0000	1.0000	0.2320
22	1.0000	1.0000	1.0000	58	0.2844	0.2649	0.2540
23	0.5210	0.3975	0.2896	59	0.7497	0.7286	0.6828
24	1.0000	1.0000	1.0000	60	1.0000	1.0000	1.0000
25	0.7744	0.7727	0.7172	61	1.0000	1.0000	1.0000
26	0.6145	0.5170	0.4984	62	0.3425	0.3330	0.3225
27	0.2606	0.2504	0.2134	63	0.5355	0.5130	0.4455
28	0.3330	0.3251	0.2913	64	1.0000	1.0000	1.0000
29	1.0000	1.0000	1.0000	65	1.0000	0.9673	0.8335
30	1.0000	1.0000	1.0000	66	0.7330	0.4449	0.2438
31	1.0000	1.0000	1.0000	67	0.3809	0.3809	0.2848
32	0.3760	0.3727	0.3686	68	1.0000	1.0000	1.0000
33	0.4065	0.4026	0.3966	69	1.0000	1.0000	1.0000
34	1.0000	1.0000	1.0000	70	1.0000	0.8081	0.3901
35	1.0000	1.0000	1.0000	71	0.2397	0.2393	0.2323
36	0.6975	0.6908	0.6679				

#### Stochastic value-based $SE$ scores

The results for both the lower and upper bounds of the stochastic value-based $SE$ scores for all 71 banks are presented in Tables 6 and 7 for $\beta =0.99$ and $\beta =1.01$, respectively.

**Table 6:** The stochastic value-based scale elasticity scores of 71 banks $\left(\beta =0.99\right)$

Banks	$\gamma =0.1$			$\gamma =0.3$			$\gamma =0.5$		
	ε^−^	ε^+^	RTS	ε^−^	ε^+^		ε^−^	ε^+^	RTS
1	1.0070	$\infty$	D	1.0109	$\infty$	D	1.0142	$\infty$	D
2	1.0484	11.4017	D	1.0533	9.5235	D	1.0576	6.6385	D
3	1.0106	6.2381	D	1.0110	6.1751	D	1.0125	5.5490	D
4	1.2171	1.2171	D	1.1296	1.1296	D	1.0012	1.0012	D
5	–	–		− 3.2891	17.3709	C	− 0.6043	7.3817	C
6	1.5299	$\infty$	D	1.7047	$\infty$	D	2.0646	$\infty$	D
7	1.0098	3.1783	D	1.0210	2.3129	D	1.0253	1.8738	D
8	0.8946	386.0003	C	0.8946	184.0351	C	0.9196	139.9671	C
9	–	–		1.1603	$\infty$	D	3.7360	$\infty$	D
10	− 0.3848	$\infty$	C	− 0.1447	$\infty$	C	0.0521	$\infty$	C
11	–	–		–	–		-	–	
12	1.4826	1.4826	D	1.5579	1.5579	D	1.0385	1.0385	D
13	1.0730	$\infty$	D	1.0776	$\infty$	D	1.0812	$\infty$	D
14	1.1860	1.1860	D	1.2406	1.2406	D	1.3145	1.3145	D
15	1.1639	10.5830	D	1.1647	7.6843	D	1.2100	5.0648	D
16	1.1293	19.9930	D	1.1335	15.9026	D	1.1501	14.3304	D
17	1.0167	6.5567	D	1.0173	6.1923	D	1.0470	5.7073	D
18	1.0466	30.4380	D	1.0740	28.0507	D	1.1022	26.5802	D
19	0.7687	$\infty$	C	0.8313	$\infty$	C	0.9020	$\infty$	C
20	0.5361	6.9435	C	0.5556	5.6046	C	0.5858	5.1075	C
21	0.5521	$\infty$	C	0.6984	$\infty$	C	0.7706	$\infty$	C
22		–		–	–		–	–	
23	2.5434	2.5434	D	1.8331	1.8331	D	1.4801	1.4801	D
24	1.0066	4.9098	D	1.0073	4.4078	D	1.0952	1.9956	D
25	1.2750	1.2750	D	1.2639	1.2639	D	1.0701	1.0701	D
26	1.4901	1.4901	D	1.3415	1.3415	D	1.0367	1.0367	D
27	1.0482	1.0482	D	1.0529	1.0529	D	1.1343	1.1343	D
28	0.9772	0.9772	I	0.9696	0.9696	I	0.9910	0.9910	I
29	1.0572	$\infty$	D	1.1094	4.7523	D	1.2502	2.1401	D
30	–	–		–	–		–	–	
31	− 0.6268	3.4159	C	− 0.5915	3.4132	C	− 0.5477	3.2967	C
32	1.1490	1.1490	D	1.1351	1.1351	D	1.1212	1.1212	D
33	1.2137	1.2137	D	1.1724	1.1724	D	1.0755	1.0755	D
34	− 2.6725	$\infty$	C	0.2401	$\infty$	C	0.4459	$\infty$	C
35	0.8935	13.1266	C	0.8939	9.0470	C	0.9215	6.2906	C
36	1.4157	1.4157	D	1.4922	1.4922	D	1.9398	1.9398	D
37	1.1977	1.1977	D	1.2772	1.2772	D	0.9729	0.9729	I
38	1.0843	1.0843	D	1.0848	1.0848	D	1.1231	1.1231	D
39	0.9801	1.5486	C	0.9871	1.5434	C	1.0063	1.5222	D
40	0.9624	1.4947	C	0.9928	1.2083	C	1.0434	1.0434	D
41	0.6076	0.6076	I	0.6242	0.6242	I	0.7192	0.7192	I
42	–	–		1.0972	1.0972	D	1.0928	1.0928	D
43	1.0018	1.0018	D	0.9012	0.9012	I	0.9093	0.9093	I
44	− 2.2609	2.5154	C	− 0.6889	0.9334	I	0.4000	0.6492	I
45	–	–		–	–		–	–	
46	1.2482	1.2482	D	1.3302	1.3302	D	1.5969	1.5969	D
47	0.8720	3.1493	C	1.1119	2.6207	D	1.5761	1.7330	D
48	–	–		–	–		–	–	
49	− 69.9568	1.5018	C	− 61.9179	1.7270	C	− 53.7488	1.8606	C
50	1.0538	1.0538	D	1.0576	1.0576	D	1.0668	1.0668	D
51	− 1.5390	3.1575	C	0.2397	2.0498	C	0.7446	0.7446	I
52	–	–		–	–		–	–	
53	1.1025	1.1025	D	1.0088	1.0088	D	1.0845	1.0845	D
54	− 56.2616	− 0.0074	I	− 55.5663	− 0.0121	I	− 54.7889	− 0.0168	I
55	1.4872	1.4872	D	1.4227	1.4227	D	1.4280	1.4280	D
56	1.8469	1.8469	D	1.5170	1.5170	D	1.4573	1.4573	D
57	–	–		0.4696	10.5619	C	1.0211	1.0211	D
58	0.8103	0.8103	I	0.7229	0.7229	I	0.8094	0.8094	I
59	0.1205	0.1205	I	0.0320	0.0320	I	− 0.2105	− 0.2105	I
60	1.0048	23.1924	D	1.0048	6.6976	D	1.0369	3.6683	D
61	–	–		–	–		$\infty$	$\infty$	C
62	0.8635	0.8635	I	0.8724	0.8724	I	0.7810	0.7810	I
63	0.9400	0.9400	I	0.9386	0.9386	I	0.9337	0.9337	I
64	–	–		–	–		–	–	
65	0.4776	0.8810	I	0.6441	0.6441	I	0.7026	0.7026	I
66	0.0877	0.0877	I	0.6432	0.6432	I	0.6943	0.6943	I
67	0.1652	0.1652	I	0.1652	0.1652	I	0.3640	0.3640	I
68	–	–		− 6.6082	0.3660	I	− 2.7329	− 0.0182	I
69	0.9598	$\infty$	C	0.9606	48.2857	C	0.9765	19.3679	C
70	1.1957	$\infty$	D	1.2744	1.2744	D	1.2085	1.2085	D
71	0.5607	0.5607	I	0.5548	0.5548	I	0.4803	0.4803	I

*RTS* Returns to scale, *I* IRS, *C* CRS, *D* DRS

**Table 7:** The stochastic value-based scale elasticity scores of 71 banks $\left(\beta =1.01\right)$

Banks	$\gamma =0.1$			$\gamma =0.3$			$\gamma =0.5$		
	ε^−^	ε^+^	RTS	ε^−^	ε^+^	RTS	ε^−^	ε^+^	RTS
1	–	–		–	–		–	–	
2	1.1224	10.4163	D	1.1061	8.8375	D	1.1014	6.3011	D
3	1.0137	5.9619	D	1.0140	5.9036	D	1.0152	5.3158	D
4	1.2119	1.2119	D	1.1267	1.1267	D	1.0012	1.0012	D
5	–	–		− 2.6940	15.5703	C	− 0.4779	7.0734	C
6	–	–		–	–		–	–	
7	1.0131	3.1929	D	1.0226	2.3752	D	1.0263	1.8924	D
8	3.0215	89.0920	D	2.0734	78.8639	D	1.4649	73.7518	D
9	–	–		–	–		–	–	
10	–	–		–	–		–	–	
11	–	–		–	–		–	–	
12	1.4685	1.4685	D	1.5409	1.5409	D	1.0377	1.0377	D
13	–	–		–	–		–	–	
14	1.1816	1.1816	D	1.2347	1.2347	D	1.3063	1.3063	D
15	1.2217	9.8184	D	1.2044	7.2693	D	1.2541	4.9562	D
16	1.3305	18.0472	D	1.2855	14.8425	D	1.2920	13.5378	D
17	1.0198	6.2966	D	1.0275	5.9595	D	1.0647	5.5147	D
18	1.1402	27.5945	D	1.1539	25.6261	D	1.1870	24.3983	D
19	–	–		–	–		–	–	
20	0.5807	6.6688	C	0.5916	5.4475	C	0.6181	4.9857	C
21	–	–		–	–		–	–	
22	–	–		–	–		–	–	
23	2.4679	2.4679	D	1.8033	1.8033	D	1.4662	1.4662	D
24	1.0101	4.7680	D	1.0104	4.3468	D	1.1098	1.9997	D
25	1.2681	1.2681	D	1.2574	1.2574	D	1.0687	1.0687	D
26	1.4758	1.4758	D	1.3324	1.3324	D	1.0360	1.0360	D
27	1.0472	1.0472	D	1.0518	1.0518	D	1.1313	1.1313	D
28	0.9776	0.9776	I	0.9702	0.9702	I	0.9912	0.9912	I
29	–	–		1.1196	4.5847	D	1.2460	2.1130	D
30	–	–		–	–		–	–	
31	− 0.5584	3.4046	C	− 0.5238	3.4018	C	− 0.4832	3.2879	C
32	1.1456	1.1456	D	1.1321	1.1321	D	1.1185	1.1185	D
33	1.2086	1.2086	D	1.1684	1.1684	D	1.0739	1.0739	D
34	–	–		–	–		–	–	
35	0.9119	11.8882	C	0.9066	8.4868	C	0.9281	6.0777	C
36	1.4041	1.4041	D	1.4778	1.4778	D	1.9043	1.9043	D
37	1.1930	1.1930	D	1.2703	1.2703	D	0.9735	0.9735	I
38	1.0825	1.0825	D	1.0829	1.0829	D	1.1204	1.1204	D
39	0.9822	1.5426	C	0.9892	1.5375	C	1.0078	1.5155	D
40	0.9646	1.4918	C	0.9937	1.2074	C	1.0426	1.0426	D
41	0.6124	0.6124	I	0.6289	0.6289	I	0.7232	0.7232	I
42	–	–		1.0951	1.0951	D	1.0908	1.0908	D
43	1.0017	1.0017	D	0.9029	0.9029	I	0.9110	0.9110	I
44	− 2.2179	2.5914	C	− 0.6758	0.9568	I	0.4139	0.6563	I
45	$\infty$	0.8628	I	$\infty$	0.8371	I	$\infty$	0.7066	I
46	1.2421	1.2421	D	1.3216	1.3216	D	1.5783	1.5783	D
47	0.8841	3.1421	C	1.1379	2.6032	D	1.5605	1.7129	D
48	–	–		–	–		–	–	
49	− 219.7403	5.0918	C	− 154.0041	4.5834	C	− 110.4810	4.0562	C
50	1.0527	1.0527	D	1.0564	1.0564	D	1.0654	1.0654	D
51	− 1.4407	3.2094	C	0.2627	2.0679	C	0.7483	0.7483	I
52	–	–		–	–		–	–	
53	1.1003	1.1003	D	1.0086	1.0086	D	1.0827	1.0827	D
54	− 102.4507	0.1322	I	− 99.5819	0.1174	I	− 96.4785	0.1028	I
55	1.4729	1.4729	D	1.4109	1.4109	D	1.4160	1.4160	D
56	1.8164	1.8164	D	1.5016	1.5016	D	1.4442	1.4442	D
57	–	–		0.5426	9.9700	C	1.0206	1.0206	D
58	0.8134	0.8134	I	0.7683	0.7683	I	0.8125	0.8125	I
59	0.1226	0.1226	I	0.0327	0.0327	I	− 0.2156	− 0.2156	I
60	1.0303	19.9213	D	1.0118	6.4833	D	1.0390	3.6271	D
61	–	–		–	–		–	–	
62	0.8658	0.8658	I	0.8746	0.8746	I	0.7844	0.7844	I
63	0.9411	0.9411	I	0.9398	0.9398	I	0.9350	0.9350	I
64	–	–		$\infty$	$\infty$	C	$\infty$	0.4563	I
65	0.4872	0.8892	I	0.6486	0.6486	I	0.7068	0.7068	I
66	0.2510	0.2510	I	0.6478	0.6478	I	0.6986	0.6986	I
67	0.1679	0.1679	I	0.1679	0.1679	I	0.3687	0.3687	I
68	–	–		− 7.0120	0.4185	I	− 2.7988	0.0006	I
69	–	–		0.9809	33.3982	C	0.9842	16.6515	C
70	–	–		1.2675	1.2675	D	1.2036	1.2036	D
71	0.5657	0.5657	I	0.5598	0.5598	I	0.4852	0.4852	I

*RTS* Returns to scale, *I* IRS, *C* CRS, *D* DRS

It is well known that scale elasticity is defined only for efficient units, and for inefficient units, this computation can be performed only at their input- or output-oriented projections. However, in our case, we considered input-oriented projections. To interpret our results for $\beta =0.99$ in Table 6, for example, for bank 20, which is efficient at all three tolerance levels, the lower-bound $SE$ score is less than 1 and its upper-bound value is more than 1, that is, ${\varepsilon }^{-}<1<{\varepsilon }^{+}$, implying that its returns to scale are constant [as per Theorem 1 (b)]. For efficient bank 54, the upper bound $SE$ scores are all less than 1 (i.e., ${\varepsilon }^{+}<1)$ at all three tolerance levels, implying that its returns to scale are increasing [as per Theorem 1 (c)]; for the remaining efficient banks such as 1, 2, and 3, since the lower bound of $SE$ is greater than 1 at all three tolerance levels (i.e., ${\varepsilon }^{+}>1),$ it exhibits a decreasing return to scale [as per Theorem 1 (a)].

Note that for some stochastically inefficient banks, such as 4, 12, 14, and 23, both lower- and upper-bound $SE$ scores are equal at all three tolerance levels, implying that their projections onto the boundary are all located on *interior* points of the facet. Of the 71 banks, we find 30 banks exhibiting DRS, 10 banks exhibiting CRS, and 11 banks exhibiting IRS, at all three tolerance levels. Two more interesting observations were made in this study. First, some banks exhibit returns to scales of different types at different tolerance levels. For example, for $\beta =0.99$ in Table 6, inefficient bank 37 (at $\gamma =0.1\mathrm{ and }0.3$) exhibits DRS. This means that its projections onto the efficient frontier are all located on points of the facet exhibiting DRS. However, the projection of this inefficient bank at $\gamma =0.5$ onto the efficient frontier exhibits IRS. Furthermore, bank 51 at $\gamma =0.1\mathrm{ and }0.3$ is both VRS- and CRS-efficient and hence exhibits CRS. However, this bank was inefficient at $\gamma =0.5$. Hence, its projection onto the efficient frontier exhibits IRS. In addition, the SE results for $\beta =1.01$ in Table 7, can be interpreted analogously.

Furthermore, for $\beta =0.99$ (Table 6), the upper-bound $SE$ scores of some banks, such as 1, 6, and 10, are unbounded. This implies that any proportional increase in output lies outside the technology set. However, in Tables 6 and 7, both lower- and upper-bounds of $SE$ s of some banks, such as 45 in Table 6 and 48 in Table 7, are indicated by a dashed line, implying that model (12) is infeasible because changing $\beta$ from 1 to 0.99 or 1.01, these units appear outside the feasible region. Therefore, program (12) becomes infeasible when there is no data available outside the feasible region to determine returns to scale (Grosskopf 1996).

#### Deterministic value-based $SE$ scores of the banks

In addition to program (17), we ran the valued-based LP (4) to compute the lower and upper bounds of the $SE$ scores of 71 banks in a deterministic environment for $\beta =0.99$ and $\beta =1.01$, which are presented in Table 8. The results show that the 38 efficient banks that are found to be fully technically efficient in a stochastic environment are also efficient, and the remaining 33 banks are inefficient. Regarding their returns-to-scale characterizations, for the case of $\beta =1.01$, we find six efficient banks (i.e., 5, 20, 31, 35, 49, and 69) exhibiting CRS. For the five efficient banks (i.e., 44, 45, 54, 64, and 68), returns to scale increase because their upper bounds of $SE$ scores are all less than unity. Finally, for some efficient banks, such as 1, 6, 9, 10, 11, 13, 19, 21, 22, 30, 34, 48, 52, and 61, both lower- and upper-bound $SE$ scores are indicated by dashed lines. Hence, as discussed in Sect. 4.3, no data are available to determine the returns-to-scale type. This is because, as we consider $\beta$ from 1 to 0.99 or 1.01, these banks appear outside the feasible region. Therefore, program (3) becomes infeasible when there is no data available outside the feasible region to determine their returns-to-scale possibilities. The remaining 13 efficient banks operate under the DRS.

**Table 8:** Deterministic value-based TE and SE scores of the banks

		$\beta =1.01$			$\beta =0.99$					$\beta =1.01$			$\beta =0.99$		
Banks	$TE$	${\varepsilon }^{-}$	${\varepsilon }^{+}$		${\varepsilon }^{-}$	${\varepsilon }^{+}$		Banks	$TE$	${\varepsilon }^{-}$	${\varepsilon }^{+}$		${\varepsilon }^{-}$	${\varepsilon }^{+}$	
1	1.0000	–	–		1.0140	$\infty$	D	37	0.0760	0.9740	0.9740	I	0.9730	0.9730	I
2	1.0000	1.1010	6.3010	D	1.0580	6.6390	D	38	0.0680	1.1200	1.1200	D	1.1230	1.1230	D
3	1.0000	1.0150	5.3160	D	1.0130	5.5490	D	39	1.0000	1.0080	1.5160	D	1.0060	1.5220	D
4	0.2930	1.0010	1.0010	D	1.0010	1.0010	D	40	0.9480	1.0430	1.0430	D	1.0430	1.0430	D
5	1.0000	− 0.478	7.0730	C	− 0.604	7.3820	C	41	0.7150	0.7230	0.7230	I	0.7190	0.7190	I
6	1.0000	–	–		2.0650	$\infty$	D	42	0.0350	1.0910	1.0910	D	1.0930	1.0930	D
7	1.0000	1.0260	1.8920	D	1.0250	1.8740	D	43	0.1470	0.9110	0.9110	I	0.9090	0.9090	I
8	1.0000	1.4650	73.752	D	0.9200	139.967	C	44	1.0000	0.4140	0.6560	I	0.4000	0.6490	I
9	1.0000	–	–		3.7360	$\infty$	D	45	1.0000	$\infty$	0.7070	I	–	–	
10	1.0000	–	–		0.0520	$\infty$	C	46	0.4370	1.5780	1.5780	D	1.5970	1.5970	D
11	1.0000	–	–		–	–		47	1.0000	1.5610	1.7130	D	1.5760	1.7330	D
12	0.6700	1.0380	1.0380	D	1.0390	1.0390	D	48	1.0000	–	–		–	–	
13	1.0000	–	–		1.0810		D	49	1.0000	–110.481	4.0560	C	–53.749	1.8610	C
14	0.5050	1.3060	1.3060	D	1.3150	1.3150	D	50	0.5070	1.0650	1.0650	D	1.0670	1.0670	D
15	1.0000	1.2540	4.9560	D	1.2100	5.0650	D	51	0.4250	0.7480	0.7480	I	0.7450	0.7450	I
16	1.0000	1.2920	13.538	D	1.1500	14.330	D	52	1.0000	–	–		–	–	
17	1.0000	1.0650	5.5150	D	1.0470	5.7070	D	53	0.1660	1.0830	1.0830	D	1.0850	1.0850	D
18	1.0000	1.1870	24.398	D	1.1020	26.580	D	54	1.0000	− 96.479	0.1030	I	− 54.79	− 0.017	I
19	1.0000	–	–		0.9020	$\infty$	C	55	0.7090	1.4160	1.4160	D	1.4280	1.4280	D
20	1.0000	0.6180	4.9860	C	0.5860	5.1080	C	56	0.3770	1.4440	1.4440	D	1.4570	1.4570	D
21	1.0000	–	–		0.7710	$\infty$	C	57	0.2320	1.0210	1.0210	D	1.0210	1.0210	D
22	1.0000	–	–		–	–		58	0.2540	0.8130	0.8130	I	0.8090	0.8090	I
23	0.2900	1.4660	1.4660	D	1.4800	1.4800	D	59	0.6830	− 0.2160	− 0.216	I	− 0.211	− 0.211	I
24	1.0000	1.1100	2.0000	D	1.0950	1.9960	D	60	1.0000	1.0390	3.6270	D	1.0370	3.6680	D
25	0.7170	1.0690	1.0690	D	1.0700	1.0700	D	61	1.0000	–	–		$\infty$	$\infty$	C
26	0.4980	1.0360	1.0360	D	1.0370	1.0370	D	62	0.3230	0.7840	0.7840	I	0.7810	0.7810	I
27	0.2130	1.1310	1.1310	D	1.1340	1.1340	D	63	0.4460	0.9350	0.9350	I	0.9340	0.9340	I
28	0.2910	0.9910	0.9910	I	0.9910	0.9910	I	64	1.0000	$\infty$	0.4560	I	–	–	
29	1.0000	1.2460	2.1130	D	1.2500	2.1400	D	65	0.8340	0.7070	0.7070	I	0.7030	0.7030	I
30	1.0000	–	–		–	–		66	0.2440	0.6990	0.6990	I	0.6940	0.6940	I
31	1.0000	− 0.483	3.2880	C	− 0.548	3.2970	C	67	0.2850	0.3690	0.3690	I	0.3640	0.3640	I
32	0.3690	1.1190	1.1190	D	1.1210	1.1210	D	68	1.0000	− 2.7990	0.0010	I	− 2.733	− 0.018	I
33	0.3970	1.0740	1.0740	D	1.0760	1.0760	D	69	1.0000	0.9840	16.652	C	0.9770	19.368	C
34	1.0000	–	–		0.4460	$\infty$	C	70	0.3900	1.2040	1.2040	D	1.2090	1.2090	D
35	1.0000	0.9280	6.0780	C	0.9220	6.2910	C	71	0.2320	0.4850	0.4850	I	0.4800	0.4800	I
36	0.6680	1.9040	1.9040	D	1.9400	1.9400	D								

*I* IRS, *C* CRS, *D* DRS

For $\beta =0.99$, some efficient banks (1, 6, 9, and 13) exhibit DRS and some efficient banks (10, 19, 21, 34, and 61) exhibit CRS. However, as pointed out earlier, the returns-to-scale types of these banks for the case of $\beta =1.01$ were not determined. Moreover, for bank 8, while returns to scale are constant for $\beta =0.99$, they decrease for $\beta =1.01$.

#### A comparison between the deterministic and stochastic methods

Regarding the comparison between deterministic and stochastic $TE$ estimates, a few observations are noteworthy. First, both deterministic and stochastic methods are in complete agreement in declaring the same 38 banks to be fully technically efficient. Second, both methods yield the same value-based $TE$ scores for banks $\gamma =0.5$. This finding is consistent with our expectation (see Theorem 2), which leads us to conclude that deterministic technology is a special case of stochastic technology for $\gamma =0.5$ in exhibiting the same $TE$ scores. Third, the stochastic $TE$ scores of inefficient states are higher than their deterministic counterparts. This finding is also expected, because the main purpose of our chance-constrained formulation is to allow the observed inputs and outputs of banks to cross the efficiency frontier, but not too often. For any given observed input–output vector, the efficiency frontier in the stochastic case is now located closer than before. Finally, the efficiency scores of banks increase with sharpening (decreasing) the tolerance level of chance constraints.

Regarding the comparison between deterministic and stochastic $SE$ estimates, we find apparent differences in their returns-to-scale characterizations. First, while the stochastic method finds efficient bank 13 exhibiting DRS at all three tolerance levels considered, the deterministic method is unable to find its returns-to-scale status for $\beta =1.01$ but finds the same DRS type for $\beta =0.99$. Second, although the returns-to-scale types of the remaining banks are the same in both deterministic and stochastic methods, the degree of returns to scale (i.e., lower and upper bounds of the SE) vary to some degree. For example, consider bank 65. For the case of $\beta =0.99,$ and $\gamma$= 0.1, the stochastic lower and upper bounds of the SE estimates suggest that a 1% increase in cost raises the output by 4.78% and 8.81%, respectively. However, for $\beta =0.99$ in the deterministic case, the lower and upper $SE$ bounds suggest that a 1% increase in cost yields the same 7.03% increase in output. Third, for $\gamma =0.5,$ the value-based $SE$ scores of banks were the same between the two methods. According to Theorem 2, this finding is not contrary to our expectations. Therefore, one can conclude that deterministic technology is again a special case of stochastic technology for $\gamma =0.5$ in exhibiting the same returns-to-scale characterization of banks.

#### $TE$ vis-à-vis ownership

We examine banks’ $TE$ performance across ownership types. The results are presented in Table 9. As shown in Table 9, public banks exhibit higher $TE$ scores than private and foreign banks at all three tolerance levels. The finding of higher efficiency accrual of public banks over private and foreign banks may be due to their long-held formal status, wherein these banks have constantly been facilitating their access to scarce resources such as credit, foreign exchange, licenses, and skilled labor, which are necessary for efficient production.

**Table 9:** A comparison of stochastic $TE$ scores of banks across ownership types

	γ = 0.1	γ = 0.3	γ = 0.5
Public	0.9161	0.9015	0.8836
Private	0.7209	0.6877	0.6466
Foreign	0.7673	0.7212	0.6115

#### Returns to scale across ownership

Finally, we examined the distribution of returns-to-scale types across ownership types. For *β* = 0.99, out of 44 public and private banks, 28 (68%) operate under DRS at a confidence level γ of 0.1. As shown in Table 10, while public sector banks mostly exhibit DRS, foreign banks exhibit IRS. Returns-to-scale types of private banks are a mix that supports all types of returns-to-scale, that is, IRS, CRS, and DRS. This may be because private banks are of two types: old and new, with the former type following the tradition of public sector banks and the latter type following that of foreign banks.

**Table 10:** A comparison of stochastic returns-to-scale types of banks across ownership types

	γ = 0.1			γ = 0.3			γ = 0.5		
Ownership	IRS	CRS	DRS	IRS	CRS	DRS	IRS	CRS	DRS
*For β* = *0.99*									
Public	0	5	17	0	6	18	0	6	18
Private	2	9	11	4	7	12	6	4	13
Foreign	9	1	4	10	2	4	10	2	5
*For β* = *1.01*									
Public	0	1	15	0	2	15	0	2	15
Private	3	8	10	5	6	12	7	3	13
Foreign	9	0	3	10	3	4	11	1	5

In the next section, we discuss the results of our illustrative empirical application of Indian banks from 1998 to 2005.

## Discussions

As observed from our empirical results, different values of $\beta$ and $\gamma$ lead to varying results.

for returns-to-scale bank types. In such cases, the relevant question arises as to how bank management makes an appropriate judgment for its scale decisions. As noted earlier, the possible values of $\beta$ are user-defined values that reflect the proportional output change, and the value-based $TE$ function $\overline{\alpha }(\beta )$ for any bank $o$ is defined for all $\beta \in [0,\widehat{\beta }]$, where $\widehat{\beta }$ is the largest proportion of its output vector ${y}_{o}$ found on ${T}^{V}$. However, the scale elasticity of any radial technically efficient bank $\left({x}_{o}, {y}_{o}\right)$ is well defined at $\beta =\alpha \left(\beta \right)=1$ as we determine its returns-to-scale status locally at the neighborhood of point $\left({x}_{o}, {y}_{o}\right)$, where a derivative is to be evaluated. Since scale elasticity is a one-sided concept, one can therefore determine a bank’s (local) returns-to-scale type at its right or left by measuring the response to outputs by considering only a one percent increase or decrease in inputs, that is, $\beta =1.01$ (right) and $\beta =0.99$ (left) at a specific confidence level.^9^

Regarding the values of tolerance level $\gamma$, a pre-determined parameter ($0<\gamma \le 0.5$) makes the confidence level of each firm large or small, that is, the confidence level that forms an ellipse around the mean value of the inputs and outputs of each firm, increase or decrease. Consequently, a bank may experience a change in the returns-to-scale status for a change in $\gamma$. Therefore, the relevant question for any bank facing uncertainty is at what level of $\gamma$ should it consider making an appropriate judgment about its scale decision. Because our proposed stochastic approach generates only expected $SE$ scores rather than distributions of random $SE$ scores, we are at odds to answer this question. Our focus in this study is, however, on ex-post-facto analysis of already affected decisions, which can have certain uses in the control aspects of bank management where evaluations of returns-to-scale performance are required. We need to address the *ex-ante* (planning) problem of how to use this knowledge to arrive at the possible value of $\gamma$, which can be used while affecting future-oriented decisions regarding whether to scale up or scale down operations.

Regarding the results concerning bank efficiency performance across ownership types, we find that public banks exhibit higher efficiency than private and foreign banks. This finding fails to provide empirical support for the property rights hypothesis that private enterprises should perform better than public enterprises, which precisely holds in a developed country where there is a strong link between the market for takeover and the efficiency of private enterprises. In India, public banks are known for their better organizational structure and greater penetration into the customer base. During the post-reform period of our study (1998–2005), government policies favored public banks in managing their expansionary activities well because of their age-old learning experience. In the absence of an active market for corporate control, substantial government ownership, and a relatively low level of technological advancement, conventional wisdom that private banks should perform better needs to be challenged.

The returns-to-scale results during our study period reveal that, while large public and old private banks mostly exhibit either DRS^10^ or CRS, small foreign and new private banks experience returns-to-scale possibilities of all types. Therefore, the finding of large public and old private banks exhibiting DRS does not bode well with the perception that regulators consider very large-size banks “too-big-to-fall,” which would encourage large or excessive risk-taking to drive bank growth. On the contrary, the finding of new small- and medium-sized private and foreign banks exhibiting IRS suggests that these banks have enough opportunity to increase output by either increasing the scale or merging with other banks to improve their performance.

## Concluding remarks

Using the CC programming method, value-based measures of efficiency and scale economies’ behavior of firms are investigated from the viewpoint of the economic theory of production in the possible presence of stochastic variability in the underlying input and output data. There is considerable variation in the results concerning both the efficiency and returns-to-scale characterizations of banks between the deterministic and stochastic models. These differences are precisely due to the existence of confidence regions for various tolerance levels of the chance constraints. However, deterministic technology is a special case of stochastic technology at a tolerance level of 0.5, exhibiting both the efficiency and returns-to-scale characterizations of firms. Regarding the key empirical findings of our illustrative application to the Indian banking industry during the post-reform period (1998–2005), we have a few key findings. First, as expected, the stochastic model generates higher efficiency scores for inefficient banks as compared to the deterministic model. Second, public banks are more efficient than private and foreign banks, which challenges the property rights hypothesis. However, this finding is not unusual because the Indian banking industry is characterized by the absence of an active market for corporate control and substantial government ownership. Third, large public and old private banks mostly exhibit either decreasing or constant returns to scale, whereas small foreign and new private banks experience either increasing or decreasing returns to scale. Finally, based on bank-specific scale properties, the management can decide on the expansion or contraction of their operations in the subsequent operation period, which provides a vision of how to change outputs for changing inputs to preserve efficiency status in the subsequent period of activities.

### Limitations and future recommendations

The proposed stochastic model requires information on the joint probability distributions of random inputs and outputs, which are generally inferred from their respective frequency distributions. Because this information is often not available in practice, we are at a loss to estimate them empirically. Given the nature of the input and output data that we deal with in this study, they are all subject to uncertainties; therefore, the requirement of availability of data on their joint probability distributions should not constrain the use of our empirically appealing stochastic model.

However, our stochastic model cannot be used freely. First, the inputs and outputs are assumed to be well approximated by a normal distribution, although they are all subject to empirical testing. Second, the use of a single-factor assumption for linear transformation requires further assumptions in the resulting LP problem that there are perfect correlations between any two inputs/outputs, or between input and output. We strongly believe that in any real-life decision-making situation characterized by the presence of uncertainty, the benefit of using the stochastic model outweighs its cost. For example, if the underlying objective is to analyze the efficiency, returns to scale, and returns to growth behavior of technology-intensive firms in the new economy, one must resort to the use of a stochastic model because these firms are all characterized by the presence of uncertainty concerning inventory holdings, excess capacity, and organizational slack as contingencies against uncertain future developments.

Finally, we point to some avenues for future potential research subjects.

- From our proposed stochastic model, the possible presence of input and output slack for individual banks can be explored. Unlike in the case of a deterministic model, the slacks here can presumably be interpreted as inventory (i.e., excess reserves), desired excess capacity, and organizational slack, which firms must hold as contingencies against uncertain future developments. This analysis will aid management in deciding the accumulation of optimal slack to sustain market competition and price uncertainty.
- One could also extend our stochastic model to network production technologies to analyze the efficiency and returns-to-scale characterizations of firms.
- Both the chance-constrained program and stochastic frontier analysis can be compared to analyze and contrast the efficiency and returns-to-scale classification of firms.
- Because the linear transformation method used in our proposed stochastic model requires the assumption of perfect correlations between inputs/outputs, the desired future research involves the development of an alternative linear transformation method, which could assume such assumptions.
- Our proposed stochastic approach yields the expected efficiencies rather than the distribution of random efficiencies. Therefore, one can use the probabilistic approach by Kao and Liu (2009, 2019) to generate distributions of efficiency and scale elasticity scores for each firm, which could be more informative for better decision-making.
- Our focus in this study is on the *ex-post-facto* analysis of already affected decisions, which can be used in the control aspects of management. However, future research requires an *ex-ante* (planning) analysis of how to use the returns to scale results based on various possible pre-defined tolerance levels to arrive at the precise tolerance level using which future-oriented decisions regarding whether to scale up or scale down operations can be affected.
- Our empirical application of 71 banks over eight years (1998–2005) is primarily for illustrative purposes. However, an extension of the data set to the year 2021 is required to conduct a detailed empirical investigation concerning whether the effects of global extreme events such as COVID-19 and internal events such as the adoption of the Insolvency and Bankruptcy Code (IBC) Bill influence banks’ efficiency and scale performance differentials. Further, since our findings are not clear as to whether scale economies still provide an impetus for banks to become larger, we expect future researchers to examine returns to scale from either revenue or profit perspectives, which can provide a more complete picture of scale economies in the Indian banking industry.
- Although the application of our proposed chance-constraint efficiency model is in banking to analyze efficiency and scale properties, it can potentially be applied to a wide selection of areas studied earlier, such as deriving innovative carbon-reducing emission strategies to increase the performance of solar energy investment projects in the transportation sector (Kou et al. 2022), evaluating the credit risks of small and medium-sized enterprises (SMEs) using payment and transactional data (Kou et al. 2021), evaluating credit ratings of online peer-to-peer (P2P) loans to control default risk and improve profit for lenders and platforms (Wang et al. 2021), evaluating the performance of various clustering algorithms that are used to assess financial risk (Kou et al. 2014), and detecting clusters in financial data to infer users’ behaviors and identify potential risks (Li et al. 2021).
- Although our current empirical application to Indian banking is illustrative, our proposed chance-constraint efficiency model can potentially be applied to analyze the efficiency and scale properties of real-life firms with stochastic underlying production processes. Examples of these firms can be found in industries such as agriculture, where unpredictability in weather makes the input–output relationship stochastic; manufacturing industry, where firms face considerable variation in the quality of their inputs and outputs produced; product development industry, where firms face uncertainty regarding their new designs; and high-technology industries, where firms face hyper (dynamic)completion in the new (Internet) economy.

## Data Availability

All data used in this paper are available per request.

## Abbreviations

DEA: Data envelopment analysis
RTS: Returns to scale
CRS: Constant returns to scale
IRS: Increasing returns to scale
DRS: Decreasing returns to scale
VRS: Variable returns to scale
SE: Scale elasticity
TE: Technical efficiency
CE: Cost efficiency
LP: Linear programming
CC: Chance-constrained

## Footnotes

1 The concepts—‘firm’, ‘decision-making unit, DMU’, and ‘production unit’—are used synonymously in this paper. However, they are conceptually different. The concept of a firm is much broader as it includes not only the technology of the production unit but also the entire gamut of organization, management, learning by doing, reorganization of inputs, and other capabilities of the firm.

2 DEA (Charnes et al. 1978; Banker et al. 1984) has also been widely used in various application areas, inter alia, economic efficiency performance of countries (Koengkan et al. 2022), resource and energy efficiency of countries (Kazemzadeh et al. 2022), human development index (Despotis 2005; Lozano and Gutierrez 2008), and strategy (Saen and Azadi 2009). See Emrouznejad et al. (2008) for an excellent survey on the scholarly literature on the evaluation of research in efficiency and productivity.

3 Even Farrell (1957) cautioned against the use of prices in determining the economic efficiency behavior of firms due to fluctuations in demand and prices (Sengupta 2000).

4 For a detailed account of how this cost efficiency measure is of limited use in real-life applications, c.f., *inter alia*, Fukuyama and Weber (2004); Camanho and Dyson (2008), Park and Cho (2011), and Sahoo et al. (2014a, b, c).

5 The stochastic production relationship in a DEA setting may arise in different situations, for example, when stochastic variations in inputs and outputs affect the production frontier; when inputs and outputs are faced with stochastic prices while measuring allocative efficiency; when the slacks obtained from the DEA efficiency frontier are analyzed in terms of their statistical distribution; when an economic method is applied to estimate the stochastic production frontier; etc. (Sengupta 1982, 1987, 1990).

6 Alternatively, one may consider using the semi-parametric stochastic frontier analysis (SFA) approach. The advantage of using this approach over the chance-constrained DEA (CC-DEA) approach is that it has a solid statistical foundation. However, the underlying problem here is that it is not well suited to accommodate the firm-specific distributions of noise and inefficiency, due to which the results may not be useful from an individual decision-making point of view in the short run. The CC-DEA approach, on the other hand, accommodates measurement error and management inefficiency in the technology by replacing the observed inputs and outputs with the firm-specific distributions of inputs and outputs.

7 To linearize the non-linear program (11), one can also use the properties of the *absolute function*.

8 Cooper et al. (2000) have suggested, in general, considering the following tolerance level of the chance constraints: $0<\gamma \le 0.5$ for which the $TE$ scores lie between 0 and 1. However, for data with large amounts of uncertainty where $0.5<\gamma <1$, the efficiency frontier moves closer to the given observations, and many observations will automatically come with unity or close to unit *TE* scores. The results of the CC program may then be questioned. That is why most of the studies in the stochastic case have considered the values for* γ* between 0 and 0.5, i.e., 0 <* γ* ≤ 0.5.

9 However, following Banker et al. (2004), one can consider evaluating the returns-to-scale type of bank $\left({x}_{o},\mathrm{ }{x}_{o}\right)$ globally with respect to the most productive scale size by jointly maximizing the proportional increase in outputs and minimizing the proportional decrease in inputs.

10 This finding is at odds with Das and Das (2007) that there is no evidence of diseconomies of scale for larger banks. We believe that this contrasting finding is due the use of econometric method, which, we suspect, can confound the effects of misspecification of functional form with scale economies. Therefore, for a more stable picture of scale economies in the Indian banking industry, we recommend future researchers to consider an examination of returns to scale from either revenue or profit perspective, which we have indicated as one of the potential future research projects.

## References

[CR1] Atici KB, Podinovski VV (2015). Using data envelopment analysis for the assessment of technical efficiency of units with different specializations: an application to agriculture. Omega.

[CR2] Banker RD (1993). Maximum likelihood, consistency and data envelopment analysis: a statistical foundation. Manage Sci.

[CR3] Banker RD, Morey RC (1986). Efficiency analysis for exogenously fixed inputs and outputs. Oper Res.

[CR4] Banker RD, Natarajan R, Cooper WW, Seiford LM, Zhu J (2004). Statistical tests based on DEA efficiency scores. Handbook on data envelopment analysis.

[CR5] Banker RD, Charnes A, Cooper WW (1984). Some models for estimating technical and scale inefficiencies in data envelopment analysis. Manage Sci.

[CR6] Banker RD, Cooper WW, Seiford LM, Thrall RM, Zhu J (2004). Returns to scale in different DEA models. Eur J Oper Res.

[CR7] Banker, R., Park, H. U., & Sahoo, B. (2022). A statistical foundation for the measurement of managerial ability. https://mpra.ub.uni-muenchen.de/111832/

[CR8] Baumol WJ, Panzar JC, Willig RD (1982). Contestable markets and the theory of industry structure.

[CR9] Berger AN (2003). The economic effects of technological progress: evidence from the banking industry. J Money Credit Bank.

[CR10] Camanho AS, Dyson RG (2005). Cost efficiency measurement with price uncertainty: a DEA application to bank branch assessments. Eur J Oper Res.

[CR11] Camanho AS, Dyson RG (2008). A generalization of the Farrell cost-efficiency measure applicable to non-fully competitive settings. Omega.

[CR12] Charnes A, Cooper WW (1959). Chance-constrained programming. Manage Sci.

[CR13] Charnes A, Cooper W (1962). Chance constraints and normal deviates. J Am Stat Assoc.

[CR14] Charnes A, Cooper WW (1963). Deterministic equivalents for optimizing and satisficing under chance constraints. Oper Res.

[CR15] Charnes A, Cooper WW (1977). Goal programming and multiple objective optimizations: Part 1. Eur J Oper Res.

[CR16] Charnes A, Cooper WW, Rhodes E (1978). Measuring the efficiency of decision-making units. Eur J Oper Res.

[CR17] Cherchye L, Rock BD, Dierynck B, Roodhooft F, Sabbe J (2013). Opening the “black box” of efficiency measurement: input allocation in multioutput settings. Oper Res.

[CR18] Cherchye L, De Rock B, Walheer B (2015). Multi-output efficiency with good and bad outputs. Eur J Oper Res.

[CR19] Cherchye L, De Rock B, Walheer B (2016). Multi-output profit efficiency and directional distance functions. Omega.

[CR20] Cook WD, Zhu J (2011). Multiple variable proportionality in data envelopment analysis. Oper Res.

[CR21] Cooper W, Huang Z, Li SX (1996). Satisficing DEA models under chance constraints. Ann Oper Res.

[CR22] Cooper WW, Huang Z, Lelas V, Li SX, Olesen OB (1998). Chance constrained programming formulations for stochastic characterizations of efficiency and dominance in DEA. J Prod Anal.

[CR23] Cooper WW, Deng H, Huang Z, Li SX (2002). Chance constrained programming approaches to technical efficiencies and inefficiencies in stochastic data envelopment analysis. J Oper Res Soc.

[CR24] Cooper WW, Deng H, Huang Z, Li SX (2004). Chance constrained programming approaches to congestion in stochastic data envelopment analysis. Eur J Oper Res.

[CR25] Cooper WW, Seiford LM, Tone K (2000) Data envelopment analysis. In: Cooper WW, Seiford LM, Zhu J (eds) Handbook on data envelopment analysis, 1st edn, pp 1–40

[CR26] Cooper WW, Huang Z, Li SX (2011) Chance-constrained DEA. In: Handbook on data envelopment analysis. Springer, Boston, MA, pp 211–240

[CR27] Das A, Das S (2007). Scale economies, cost complementarities and technical progress in Indian banking: evidence from fourier flexible functional form. Appl Econ.

[CR28] De Alessi L, Richard OZ (1980). The economics of property rights: a review of the evidence. Research in law and economics: a research annual.

[CR29] Despotis DK (2005). A reassessment of the human development index via data envelopment analysis. J Oper Res Soc.

[CR30] Emrouznejad A, Parker BR, Tavares G (2008). Evaluation of research in efficiency and productivity: a survey and analysis of the first 30 years of scholarly literature in DEA. Socioecon Plann Sci.

[CR31] Färe R, Grosskopf S, Lovell CAK (1985). The measurement of efficiency of production.

[CR32] Färe R, Grosskopf S, Lovell CAK (1988). Scale elasticity and scale efficiency. J Inst Theor Econ.

[CR33] Farrell MJ (1957). The measurement of productive efficiency. J R Stat Soc.

[CR34] FarzipoorSaen R, Azadi M (2009). The use of super-efficiency analysis for strategy ranking. Int J Soc Syst Sci.

[CR35] Førsund FR (1996). On the calculation of the scale elasticity in DEA models. J Prod Anal.

[CR36] Fukuyama H, Weber WL (2004). Economic inefficiency measurement of input spending when decision-making units face different input prices. J Oper Res Soc.

[CR37] Fukuyama H, Weber WL (2008). Profit inefficiency of Japanese securities firms. J Appl Econ.

[CR38] Fusco E, Vidoli F, Sahoo BK (2018). Spatial heterogeneity in composite indicator: a methodological proposal. Omega.

[CR39] Grosskopf S (1996). Statistical inference and nonparametric efficiency: a selective survey. J Prod Anal.

[CR40] Huang Z, Li SX (1996). Dominance stochastic models in data envelopment analysis. Eur J Oper Res.

[CR41] Huang Z, Li SX (2001). Stochastic DEA models with different types of input-output disturbances. J Prod Anal.

[CR42] Jess A, Jongen HT, Neralić L, Stein O (2001). A semi-infinite programming model in data envelopment analysis. Optimization.

[CR43] Kahane Y (1977). Determination of the product mix and the business policy of an insurance company—a portfolio approach. Manag Sci.

[CR44] Kao C, Liu ST (2009). Stochastic data envelopment analysis in measuring the efficiency of Taiwan commercial banks. Eur J Oper Res.

[CR45] Kao C, Liu ST (2014). Measuring performance improvement of Taiwanese commercial banks under uncertainty. Eur J Oper Res.

[CR46] Kao C, Liu ST (2019). Stochastic efficiency measures for production units with correlated data. Eur J Oper Res.

[CR47] Kazemzadeh E, Fuinhas JA, Koengkan M, Osmani F, Silva N (2022). Do energy efficiency and export quality affect the ecological footprint in emerging countries? A two-step approach using the SBM–DEA model and panel quantile regression. Environ Syst Decis.

[CR48] Koengkan M, Fuinhas JA, Kazemzadeh E, Osmani F, Alavijeh NK, Auza A, Teixeira M (2022). Measuring the economic efficiency performance in Latin American and Caribbean countries: an empirical evidence from stochastic production frontier and data envelopment analysis. Int Econ.

[CR49] Kou G, Peng Y, Wang G (2014). Evaluation of clustering algorithms for financial risk analysis using MCDM methods. Inf Sci.

[CR50] Kou G, Xu Y, Peng Y, Shen F, Chen Y, Chang K, Kou S (2021). Bankruptcy prediction for SMEs using transactional data and two-stage multiobjective feature selection. Decis Support Syst.

[CR51] Kou G, Yüksel S, Dinçer H (2022). Inventive problem-solving map of innovative carbon emission strategies for solar energy-based transportation investment projects. Appl Energy.

[CR110] Kuosmanen T (2005). Weak disposability in nonparametric production analysis with undesirable outputs. Am J Agric Econ.

[CR52] Lamb JD, Tee KH (2012). Resampling DEA estimates of investment fund performance. Eur J Oper Res.

[CR53] Land KC, Lovell CAK, Thore S (1993). Chance-constrained data envelopment analysis. Manag Decis Econ.

[CR54] Land KC, Lovell CAK, Thore S (1994). Productive efficiency under capitalism and state socialism: an empirical inquiry using chance-constrained data envelopment analysis. Technol Forecast Soc Chang.

[CR55] Levy B (1987). A theory of public enterprise behavior. J Econ Behav Organ.

[CR56] Li SX (1995). A satisficing chance constrained model in the portfolio selection of insurance lines and investments. J Oper Res Soc.

[CR57] Li SX (1995). An insurance and investment portfolio model using chance constrained programming. Omega.

[CR58] Li SX (1998). Stochastic models and variable returns to scales in data envelopment analysis. Eur J Oper Res.

[CR59] Li SX, Huang Z (1996). Determination of the portfolio selection for a property-liability insurance company. Eur J Oper Res.

[CR60] Li T, Kou G, Peng Y, Philip SY (2021) An integrated cluster detection, optimization, and interpretation approach for financial data. IEEE Trans Cybern 10.1109/TCYB.2021.3109066 34550896

[CR61] Lozano S, Gutiérrez E (2008). Data envelopment analysis of the human development index. Int J Soc Syst Sci.

[CR62] Morita H, Seiford LM (1999). Characteristics on stochastic DEA efficiency. J Oper Res Soc.

[CR63] Niskanen WA (1975). Bureaucrats and politicians. J Law Econ.

[CR64] Olesen OB (2006). Comparing and combining two approaches for chance-constrained DEA. J Prod Anal.

[CR65] Olesen OB, Petersen N (1995). Chance constrained efficiency evaluation. Manag Sci.

[CR66] Olesen OB, Petersen NC (2016). Stochastic data envelopment analysis—a review. Eur J Oper Res.

[CR67] Olesen OB, Petersen NC (2000) Foundation of chance constrained data envelopment analysis for Pareto-Koopmann efficient production possibility sets. In: International DEA symposium 2000, measurement and improvement in the 21st century. The University of Queensland, pp 313–349

[CR68] Panzar JC, Willig RD (1977) Economies of scale in multi-output production. Quart J Econ, 481–493

[CR69] Park KS, Cho J-W (2011). Pro-efficiency: data speak more than technical efficiency. Eur J Oper Res.

[CR70] Podinovski VV (2004). Production trade-offs and weight restrictions in data envelopment analysis. J Oper Res Soc.

[CR71] Podinovski VV (2004). Bridging the gap between the constant and variable returns-to-scale models: selective proportionality in data envelopment analysis. J Oper Res Soc.

[CR72] Podinovski VV (2007). Improving data envelopment analysis by the use of production trade-offs. J Oper Res Soc.

[CR73] Podinovski VV (2016). Optimal weights in DEA models with weight restrictions. Eur J Oper Res.

[CR74] Podinovski VV, Førsund FR, Krivonozhko VE (2009). A simple derivation of scale elasticity in data envelopment analysis. Eur J Oper Res.

[CR75] Podinovski VV, Chambers RG, Atici KB, Deineko ID (2016). Marginal values and returns to scale for nonparametric production frontiers. Oper Res.

[CR76] Podinovski, V. V. (2015). DEA models with production trade-offs and weight restrictions. In: Data envelopment analysis. Springer, Boston, pp 105–144.

[CR77] Sahoo BK, Acharya D (2010). An alternative approach to monetary aggregation in DEA. Eur J Oper Res.

[CR78] Sahoo BK, Gstach D (2011). Scale economies in Indian commercial banking sector: evidence from DEA and translog estimates. Int J Inf Syst Soc Chang.

[CR79] Sahoo B, Sengupta J (2014). Neoclassical characterization of returns to scale in nonparametric production analysis. J Quant Econ.

[CR80] Sahoo BK, Tone K (2009). Decomposing capacity utilization in data envelopment analysis: an application to banks in India. Eur J Oper Res.

[CR81] Sahoo BK, Tone K (2009). Radial and non-radial decompositions of profit change: with an application to Indian banking. Eur J Oper Res.

[CR82] Sahoo BK, Tone K (2013). Non-parametric measurement of economies of scale and scope in non-competitive environment with price uncertainty. Omega.

[CR83] Sahoo BK, Tone K (2022). Evaluating the potential efficiency gains from optimal industry configuration: a case of life insurance industry of India. Manag Decis Econ.

[CR84] Sahoo BK, Mohapatra PKJ, Trivedi ML (1999). A comparative application of data envelopment analysis and frontier translog production function for estimating returns to scale and efficiencies. Int J Syst Sci.

[CR85] Sahoo BK, Kerstens K, Tone K (2012). Returns to growth in a nonparametric DEA approach. Int Trans Oper Res.

[CR86] Sahoo BK, Mehdiloozad M, Tone K (2014). Cost, revenue and profit efficiency measurement in DEA: a directional distance function approach. Eur J Oper Res.

[CR87] Sahoo BK, Zhu J, Tone K, Klemen BM (2014). Decomposing technical efficiency and scale elasticity in two-stage network DEA. Eur J Oper Res.

[CR88] Sahoo BK, Singh R, Mishra B, Sankaran K (2017). Research productivity in management schools of India during 1968–2015: a directional benefit-of-doubt model analysis. Omega.

[CR89] Sahoo BK, Tone K (2015) Scale elasticity in non-parametric DEA approach. In: Data envelopment analysis. Springer, Boston, pp 269–290

[CR90] Sahoo BK, Zhu J, Tone K (2014a) Decomposing efficiency and returns to scale in two-stage network systems. In: Data envelopment analysis. Springer, Boston, pp 137–164

[CR91] Sengupta JK (1982). Efficiency measurement in stochastic input-output systems. Int J Syst Sci.

[CR92] Sengupta JK (1987). Data envelopment analysis for efficiency measurement in the stochastic case. Comput Oper Res.

[CR93] Sengupta JK (1990). Transformations in stochastic DEA models. J Econom.

[CR94] Sengupta JK (2000). Dynamic and stochastic efficiency analysis: economics of data envelopment analysis.

[CR95] Sengupta JK, Sfeir RE (1988). Minimax method of measuring productive efficiency. Int J Syst Sci.

[CR96] Sharpe WF (1963). A simplified model for portfolio analysis. Manage Sci.

[CR97] Shiraz RK, Hatami-Marbini A, Emrouznejad A, Fukuyama H (2018). Chance-constrained cost efficiency in data envelopment analysis model with random inputs and outputs. Oper Res.

[CR98] Simar L, Wilson PW (2015). Statistical approaches for non-parametric frontier models: a guided tour. Int Stat Rev.

[CR99] Sueyoshi T (1997). Measuring efficiencies and returns to scale of Nippon telegraph & telephone in production and cost analyses. Manag Sci.

[CR100] Tone K (2001). On returns to scale under weight restrictions in data envelopment analysis. J Prod Anal.

[CR101] Tone K (2002). A strange case of the cost and allocative efficiencies in DEA. J Oper Res Soc.

[CR102] Tone K, Sahoo BK (2003). Scale, indivisibilities and production function in data envelopment analysis. Int J Prod Econ.

[CR103] Tone K, Sahoo BK (2004). Degree of scale economies and congestion: a unified DEA approach. Eur J Oper Res.

[CR104] Tone K, Sahoo BK (2005). Evaluating cost efficiency and returns to scale in the Life Insurance Corporation of India using data envelopment analysis. Socioecon Plann Sci.

[CR105] Tone K, Sahoo BK (2006). Re-examining scale elasticity in DEA. Ann Oper Res.

[CR106] Wang H, Kou G, Peng Y (2021). Multi-class misclassification cost matrix for credit ratings in peer-to-peer lending. J Oper Res Soc.

[CR107] Wei G, Chen J, Wang J (2014). Stochastic efficiency analysis with a reliability consideration. Omega.

